# Fibrous Flagellar Hairs of *Chlamydomonas reinhardtii* Do Not Enhance Swimming

**DOI:** 10.1016/j.bpj.2020.05.003

**Published:** 2020-05-19

**Authors:** Guillermo J. Amador, Da Wei, Daniel Tam, Marie-Eve Aubin-Tam

**Affiliations:** 1 Laboratory for Aero and Hydrodynamics, Delft University of Technology, Delft, the Netherlands; 2 Department of Bionanoscience, Kavli Institute of Nanoscience, Delft University of Technology, Delft, the Netherlands

## Abstract

The flagella of *Chlamydomonas reinhardtii* possess fibrous ultrastructures of a nanometer-scale thickness known as mastigonemes. These structures have been widely hypothesized to enhance flagellar thrust; however, detailed hydrodynamic analysis supporting this claim is lacking. In this study, we present a comprehensive investigation into the hydrodynamic effects of mastigonemes using a genetically modified mutant lacking the fibrous structures. Through high-speed observations of freely swimming cells, we found the average and maximum swimming speeds to be unaffected by the presence of mastigonemes. In addition to swimming speeds, no significant difference was found for flagellar gait kinematics. After our observations of swimming kinematics, we present direct measurements of the hydrodynamic forces generated by flagella with and without mastigonemes. These measurements were conducted using optical tweezers, which enabled high temporal and spatial resolution of hydrodynamic forces. Through our measurements, we found no significant difference in propulsive flows due to the presence of mastigonemes. Direct comparison between measurements and fluid mechanical modeling revealed that swimming hydrodynamics were accurately captured without including mastigonemes on the modeled swimmer’s flagella. Therefore, mastigonemes do not appear to increase the flagella’s effective area while swimming, as previously thought. Our results refute the longstanding claim that mastigonemes enhance flagellar thrust in *C. reinhardtii*, and so, their function still remains enigmatic.

## Significance

Eukaryotic flagella are generally modeled as cylindrical and smooth, but surface structures are common and can significantly affect motility. *Chlamydomonas reinhardtii* has long served as the model organism for studies on the structure, assembly, and function of eukaryotic flagella. Decades ago, their flagella were observed to possess nanometer-thick fibers known as mastigonemes or flagellar hairs. Based on evidence from another alga *Ochromonas*, these structures have been widely hypothesized to increase the effective area of flagella and enhance thrust during swimming. However, in this study, we refute this hypothesis. Specifically, we show that they do not increase swimming speed, alter flagellar deformations, or increase hydrodynamic thrust. Therefore, their function remains enigmatic but seemingly important, considering they are actively replaced after removal.

## Introduction

Hair-like, or fibrous, structures are ubiquitous features in biology. They are present in organisms ranging from plants to mammals, birds, and insects. Their functions range from temperature control (1) to sensing (2), cleaning (3), adhering (4), flying (5,6), and feeding (7,8). A number of these functions rely on hydrodynamic interactions between the fibrous structures and the surrounding fluid. The structures provide a resistance to fluid flow, and this resistance has been exploited by various organisms, e.g., to enhance the spatial resolution of chemoreception in moth antennae (9), to minimize evaporation from and deposition to mammalian (10) and insect (11) eyes, to capture food particles in aquatic filter feeders (7), to entrain viscous nectar in pollinators (8), and to generate aerodynamic lift in flapping flight of birds (12) and insects (6). Fluid-structure interactions thus appear to play a crucial role in the specialized function of certain fibrous structures.

In addition to multicellular organisms, single-celled eukaryotes have been observed to possess nanometer-scale hair-like fibers, or mastigonemes (13, 14, 15, 16, 17). Mastigonemes are present on the flagella, and so are also referred to as flagellar hairs (18). Because these organisms swim via drag-based interactions between their solid flagella and fluid-filled environments using wavelike stroke patterns (19), their mastigonemes are expected to affect swimming performance through hydrodynamic interactions.

Previous studies have shown significant contributions to drag from rigid mastigonemes on the anterior flagellum of the golden algae *Ochromonas* (20, 21, 22). These algae swim using a traveling wave through their anterior flagellum. Using both theoretical (20) and numerical (22) modeling, these studies determined that mastigonemes with a particular stiffness are necessary to capture the swimming speed and direction observed experimentally (21). If the mastigonemes are too flexible, then the models predict they would bend and their contribution to the overall drag of the flagellum would be negligible; however, above a certain stiffness, they contribute significantly. In fact, in these swimmers, the hydrodynamic effect of mastigonemes is so significant that swimming direction is contrary to the expected direction for a flagellum without mastigonemes (19). A similar thrust reversal was observed in artificial helical microswimmers with rigid mastigoneme-like structures (23).

**Figure 1:**
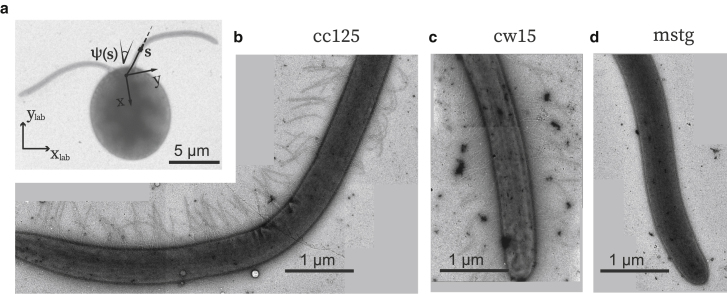
Microscopy of flagella with and without mastigonemes. (*a*) TEM image of wild-type (*cc125*) *C. reinhardtii*. (*b*–*d*) Representative TEM images of flagella from (*b*) *cc125*, (*c*) cell-wall-deficient strain (*cw15*), and (*d*) cell-wall-deficient strain without mastigonemes (*mstg*). The *cc125* and *cw15* cells clearly exhibit mastigonemes, whereas the *mstg* cells do not.

Microscopic swimmers vary significantly in morphology and swimming gaits. In swimmers in which the cell body and undulating filament are one and the same, such as *Caenorhabditis elegans*, an increase in thickness would result in a significant decrease in swimming speed (24). However, when an undulating flagellum is pushing a cell body, a thicker flagellum can lead to an increase in swimming speed (25). Besides undulating a posterior flagellum, another common swimming strategy observed in eukaryotic cells is the use of two anterior flagella. These two flagella beat in a synchronous pattern akin to a human swimmer’s breaststroke. Such a swimming gait is exhibited by the organism used in this study, *Chlamydomonas reinhardtii* (Fig. 1 *a*). The flagella of *C. reinhardtii* cells were inherited from the common ancestor of land plants and animals; as such, this organism has long served as the model organism used for studies of the structure, assembly, and function of eukaryotic flagella (26).

The mastigonemes of *C. reinhardtii* are of the fibrous type, as opposed to the tubular mastigonemes found in other algae (18) such as *Ochromonas*. Whereas tubular mastigonemes are thicker and their hydrodynamics have been studied extensively (20,21), studies into the hydrodynamics of fibrous mastigonemes are lacking, despite the fact that their bearer, *C. reinhardtii*, has drawn much attention and its swimming kinematics are well characterized (27, 28, 29, 30, 31, 32, 33).

Following their initial observation and characterization (15), the mastigonemes of *C. reinhardtii* (shown in Fig. 1 *b*) were widely hypothesized to enhance hydrodynamic performance of flagella by increasing the flagella’s effective area (18,34) because of their long length and high density. However, experimental evidence supporting their hydrodynamic function has been limited to the observation of a decrease in swimming speed of 20–30% for *C. reinhardtii* cells treated with mAb-MAST1 (35), a monoclonal antibody that was reported to trigger the loss of mastigonemes. Because this is the only experimental observation on the hydrodynamics of fibrous mastigonemes, and other information such as their effects on gait kinematics and drag force are lacking, we were motivated to gather more detailed knowledge in this regard. We thus conducted our research to resolve the function of these ultrastructures at the single-cell level.

In this study, we used a genetically modified mutant that possesses no mastigonemes (36), and therefore avoid using an antibody to remove mastigonemes. This mutant, referred to as *mstg*, is generated via insertional mutagenesis in which the mutation is inserted into the gene predicted to code for a mastigoneme protein (GenBank: AF508983, MST1) (37). The parent strain used for mutation is *cw15*, a cell-wall-deficient strain of the wild-type *C. reinhardtii*. Although *cw15* lacks a cell wall (38), it still possesses mastigonemes on its flagella. For completeness, we also included the extensively characterized wild-type strain cc-125 mt + as another control group and denote it as *cc125*. Through a thorough comparative study in the cells’ free-swimming dynamics, flagellar kinematics, and flagellar hydrodynamics across the three strains (*mstg*, *cw15*, and *cc125*), we investigate the potential hydrodynamic effects of mastigonemes.

We first assessed the presence of mastigonemes in the flagella of the three strains of *C. reinhardtii* with transmission electron microscopy (TEM). Through these observations, we confirmed the presence of mastigonemes in *cc125* and *cw15*, and their absence in the *mstg* mutant. We then analyzed the trajectories of freely swimming cells. In these observations we compared the swimming speeds, beating frequencies, and turning rates to determine how mastigonemes affect locomotory performance and kinematics. Then, we compared the flagellar gait kinematics with high-speed videography of captured single cells to determine how they are affected by the removal of the cell wall or mastigonemes. Next, we performed velocimetry measurements using optical-tweezers-based flow velocimetry (OTV), following the protocol in (33). The OTV technique measures the displacement of an optically trapped particle within the laser, from which fluid force and velocity are inferred directly. Our technique provides high temporal resolution to characterize the hydrodynamic effects of the mastigonemes near a captured algal cell. Finally, following our measurements, we compared our experimental results to numerical simulations that solve Stokes equations around a beating cell assuming smooth (hairless) flagella.

## Materials and Methods

### Cells and cell culture

Three strains of *C. reinhardtii* were used in experiments: wild-type *cc125* (cc-125 mt+), cell-wall-deficient *cw15* (cc-4453 mt−), and cell-wall- and mastigoneme-deficient *mstg*. The *mstg* mutant, LMJ.RY0402.136134, was generated by the Chlamydomonas Library Project using *cw15* as the parent strain (36). The *cw15* and *mstg* strains obtained from the Chlamydomonas Resource Center were propagated in the laboratory conditions over several months for acclimation to temperature and humidity.

All three strains were grown in identical conditions following established protocols, see (39). Specifically, they were cultured in Tris-minimal medium (pH 7) with sterile air bubbling. The cultures were subjected to light/dark (14:10 h) cycles with light intensity of 230 *μ*E m^-2^ s^-1^. They were harvested on the fourth day after inoculating the liquid culture when it reached a density of ∼$2\times 10^{5}$ cells mL^-1^ and then diluted in fresh Tris-minimal medium (pH 7).

### Transmission electron microscopy of flagella and mastigonemes

TEM observations were done with a JEM-1400 (JEOL, Tokyo, Japan). Liquid cultures of *cc125*, *cw15*, and *mstg* were simultaneously harvested and washed with fresh Tris-minimal medium. Thereafter, we centrifuged the cells (600 × *g* for 5 min) twice to increase the cell concentration.

Immediately after the concentrated cell suspensions were obtained, a droplet was placed on a carbon-coated copper TEM grid for a duration of 2 min. Afterwards, the excess cell suspension was removed by blotting with a filter paper, and the grids were stained by immersion in 2% uranium acetate during 1 min. We only recorded the cells with clearly attached flagella and with no sign of cell lysis.

### High-speed imaging and light microscopy

For our observations of freely swimming and captured cells, we used an inverted microscope (Nikon Eclipse Ti-U; Nikon, Tokyo, Japan) with a 60× water immersion objective (Nikon CFI Plan Apo VC 60× NA = 1.20; Nikon). The high-speed videography was conducted using a sCMOS high-speed camera (PCO.edge; PCO Tech, Kelheim, Germany). We obtained a spatial resolution of 0.1 *μ*m px^-1^and temporal resolution of 301 and 699 Hz for observations of freely swimming and captured cells, respectively.

All cells were observed in conditions similar to those in Wei et al. (33) and Quaranta et al. (39). Cells suspended in Tris-minimal medium were placed within a custom flow chamber (20 mm wide by 2 mm high). The chamber was sealed using silicone oil to ensure no background flows were present due to fluid evaporation. Before every experiment, suspended particles or cell debris were observed to confirm the absence of background flows. The flow chamber was fixed onto a piezoelectric stage (Nano-Drive; Mad City Labs, Madison, WI) integrated on the inverted microscope. For the free-swimming experiments, the cells were then observed directly using high-speed videography.

For the flagellar kinematics and OTV experiments, the cells were held by micropipettes made of borosilicate glass. The micropipette was mounted onto a micromanipulator (SYS-HS6; World Precision Instruments, Sarasota, FL).

### Analysis of freely swimming cells

Frame rates of the videography for free-swimming observations were set at 301 Hz, which was sufficient to detect the cells’ motion and resolve their flagella beating at $f_{0}\approx 50$ Hz. The focal plane was placed at a height of 100 *μ*m above the bottom of the flow chambers (to match our OTV measurements), and swimming tracks within ± ∼30 *μ*m of the focal plane with minimal visual variation in the vertical axis were collected.

**Figure 2:**
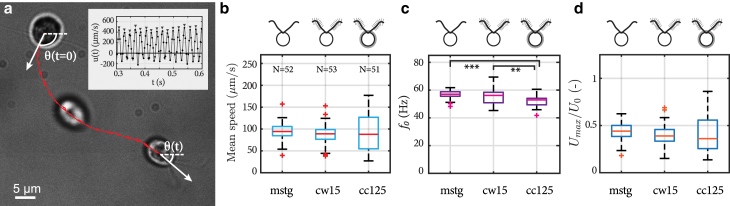
Kinematics of freely swimming cells. (*a*) Overlaid image sequence from a typical observation. The time interval between the overlaid snapshots is 200 ms. The red line represents the position of the center of the tracked cell. The white arrows depict the heading angle *θ* of the cell. The inset shows the instantaneous velocity for the cell in the image with the maximum velocities highlighted by the triangles. (*b*–*d*) Comparison of (*b*) mean swimming speed, (*c*) beating frequency $f_{0}$, and (*d*) maximum swimming speed $U_{max}$ for *mstg*, *cw15*, and *cc125* cells from left to right, respectively. (*d*) $U_{max}$ represents the median of the peak velocities depicted in the inset of (*a*) and is normalized by $U_{0}=Lf_{0}$, where *L* is flagella length. In (*c*), ^∗∗^ represents p<0.01 and ^∗∗∗^ represents p<0.001. The distributions in (*b*–*d*) are compared using the Kruskal-Wallis one-way ANOVA statistical test. For (*b*–*d*), and all distributions in the subsequent figures, the red lines represent the medians and the bottom and top of the boxes the 25th and 75th interquartiles, respectively. The whiskers represent data between $q_{1}-1.5\left(q_{3}-q_{1}\right)$ to $q_{3}+1.5\left(q_{3}-q_{1}\right)$, where $q_{1}$ and $q_{3}$ are the 25th and 75th interquartiles, respectively. Any points outside of this range are marked as red crosses and considered outliers. To see this figure in color, go online.

Our results consisted of *N* = 51, 53, and 52 tracks of the *cc125*, *cw15*, and *mstg* strains, respectively, with a mean track duration of 0.9 s or 45 beat cycles. From the videos, centers of the cell body were tracked, as shown in Fig. 2 *a*.

We calculated the mean and the maximum advancing speed for each track. Instantaneous velocity for each track (*inset* Fig. 2 *a*) was used to find the maximum advancing speed $U_{max}$. $U_{max}$ was the median of the peak velocities, as highlighted by the triangles in the inset of Fig. 2 *a*.

For the mean speed, we discarded the oscillatory part of the track, which corresponds to the back-and-forth component of motion, by applying a low-pass filter (<10 Hz), and calculated the mean speed as the arc length of the resulting trajectory divided by the track duration.

To find the beating frequency $f_{0}$, we used two methods involving the fast Fourier transformation (FFT) function in MATLAB (The MathWorks, Natick, MA). The first method has been used in previous studies to resolve the beating frequency of both single cells and populations of cells (40, 41, 42, 43). This method uses visual vibration of the cell body in the video as input. Practically, sums of pixel value of each frame were first used to construct a time series, which records the bodily vibrations directly resulting from flagellar beating, and $f_{0}$ was resolved from the FFT of the time series. To highlight the informative part, we applied a tracking mask that blacked out all but a circular region of radius of ∼8 *μ*m around the cell center and kept the spectrum higher than 10 Hz for frequency analysis. The second method used FFT of the tracks of the cell centers (*red curve* in Fig. 2 *a*). In the frequency domain, the peaks represented the rate at which the cell body proceeded back and forth along the track. For each track, the sharpest peak between the two methods was reported as $f_{0}$ (Fig. 2 *c*). The two methods made use of different input information, namely, the flagellar-beating-induced fluctuation in the cell’s shading and the flagellar-beating-induced locomotion. When an observed cell is slightly out of focus or the net forward motion is not substantial, then the FFT of the image results in a sharper peak of $f_{0}$. On the other hand, when the cell is in focus and has substantial forward motion, then the FFT of the track results in a sharper peak. However, results of the two methods corroborate each other well.

The turning rate was calculated using the cells’ heading direction *θ*, depicted by the white arrows in Fig. 2 *a*. *θ* was found from the smoothed trajectory used for the mean speed calculations (using the <10-Hz low-pass filter). The smoothed trajectory did not contain the cells’ back-and-forth motion. The turning rate was then calculated as the average of the absolute value of the rate of change of the cells’ heading direction or $\bar{\left|d\theta /dt\right|}$.

### Optical tweezers-based velocimetry

To measure the flow fields around beating cells, we used an OTV method following Wei et al. (33). This method measures the hydrodynamic force on an optically trapped bead in the vicinity of a beating cell. The optical tweezers used in the study are similar to those in Lang et al. (44). Namely, a high-powered laser (1064-nm wavelength) was focused through a water immersion objective (CFI Plan Apo VC 60× NA = 1.20; Nikon) to generate a trapping force F=−kΔx, where Δx is the bead displacement from the center of the trapping laser and *k* is the trap stiffness. The bead displacement Δx was measured at a sampling frequency of 10 kHz using back focal plane interferometry. The beads had radii of either *a* = 1 or 2.5 *μ*m, with trap stiffness values of *k* = 12–50 pN *μ*m^–1^.

**Figure 4:**
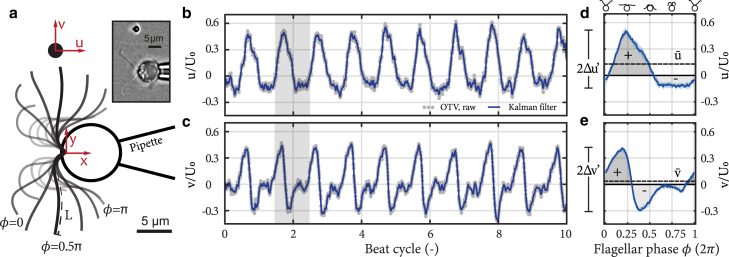
Optical tweezers-based velocimetry (OTV). (*a*) Schematic of the OTV measurements with an algal cell captured by a micropipette and optically trapped bead to measure flow velocities *u* and *v*. Inset shows a light microscope image from a typical experiment. Scale bars, 5 *μ*m. (*b* and *c*) Periodic signals of (*b*) axial *u* and (*c*) lateral *v* flow velocities as a function of beat cycle. The gray points are raw OTV data, and the blue solid curve is data filtered with a Kalman filter. A typical beat cycle is shaded; it begins with the most forward-reaching flagellar shapes, defined as flagellar phase ϕ=0, shown in the inset of (*a*).The median cycle is constructed based on ∼50 cycles and shown in (*d* and *e*). (*d* and *e*) Flow velocities (*d*) *u* and (*e*) *v* over one average beat cycle. In (*d*), the dark gray “+” represents the power stroke, when propulsive flows are generated. The light gray “−” represents the recovery stroke when the flagella move opposite to the swimming direction. In (*e*), the + and −, together with their shadings, mark, respectively, the propulsion outwards and inwards in the cell’s lateral direction. The flow amplitudes *Δu*′ and *Δv*′ are depicted on the left. The dashed lines represent the average velocities $\bar{u}$ and $\bar{v}$. The data shown in (*b*)–(*e*) are from a *cc125* cell. To see this figure in color, go online.

The flow velocity u(t)=(u,v) at the location of the trapped bead (Fig. 4 *a*) was determined from the measured bead displacement Δx following the Boussinesq-Basset-Oseen equation. Because the particle Reynolds number$Re_{a}=\frac{\rho a\left|u\right|}{\mu }\approx 10^{-5}-10^{-4}$ is low and inertia, added mass, and Basset forces are negligible (45), the Boussinesq-Basset-Oseen equation can be reduced to a first-order equation in which the trapping force F and hydrodynamic drag are balanced, or $\dot{\Delta x}+\frac{k}{\zeta }\Delta x=u\left(t\right)$, where ζ=6πμa. Similar methods have been used in previous studies to measure flows in microfluidic devices (46, 47, 48, 49).

### Numerical simulations

To model the flow fields around individual beating cells, we solved the three-dimensional (3D) Stokes equations using a hybrid boundary element method (BEM) and slender-body approach, similar to Wei et al. (33) and Kim and Karrila (45). The Stokes equations are ∇⋅u=0 and $-\nabla p+\mu \nabla^{2}u=0$, where *p* is the pressure field. These equations have been found to accurately depict the flow dynamics close to the cell (y<0.2δ, where $\delta =\sqrt{\nu /f_{0}}$ and *ν* is the kinematic viscosity of water) (33), and so, we conducted our simulations within this range.

To capture the fluid-structure interactions for the cell body and glass micropipette, we represented them with a completed double-layer boundary-integral equation (50,51). The singularities of the completion flow were distributed along the centerline of the micropipette. The flagella were represented using slender-body theory with 26 discrete points along each of the flagella’s centerline (52). The time dependent motion of each of the 26 discrete points on a beating flagellum were tracked from high-speed videos (∼700 Hz), as discussed in High-speed imaging and light microscopy. We then obtained the simulated flow field $u_{s}\left(t\right)=\left(u_{s},v_{s}\right)$, which we compared with the OTV measurements u(t)=(u,v).

## Results and Discussion

### Transmission electron microscopy of flagella and mastigonemes

The *mstg* mutant used in this study carries a mutation in the MST1 gene coding for a mastigoneme-like flagellar protein. TEM was used to confirm the presence of mastigonemes in the *cc125* and *cw15* cells, and their lack in *mstg* cells. Fig. 1, *b*–*d* show representative TEM images of flagella from *cc125*, *cw15*, and *mstg* cells, respectively. From these images we measured the flagella length L=12.0±1.7 *μ*m (average ± standard deviation, same for the rest of the text) and flagella radius r=0.23±0.02 *μ*m. The flagella from *cc125* and *cw15* exhibit two rows of mastigonemes, and the *mstg* flagella appear smooth without any mastigonemes. The mastigonemes on the *cc125* and *cw15* cells appear to be $l_{m}=850\pm 97$ nm in length and $d_{m}=16\pm 3$ nm in diameter, in agreement with previous observations (18), and with densities of 10.1±0.8 mastigonemes per micrometer. These measured values may not accurately represent the physical dimensions of the mastigonemes because the delicate structures may be affected by the staining procedure.

We observed mastigonenemes in 27 out of 28 *cc125* cells and 20 out of 21 *cw15* cells. In contrast, we found mastigonemes for none of the 22 *mstg* cells observed. Moreover, after ∼10 times of slant propagation over 18 months, during which the experiments were conducted, we repeated the comparison to confirm whether the genetic mutation was stable. Consistently, all *cc125* (N=6) and *cw15* cells (N=6) showed mastigonemes, whereas none of the *mstg* cells (N=6) did. Therefore, we conclude that the mutation of the MST1 gene was effective and stable.

Using the TEM measurements, we can estimate the possible contribution of these mastigonemes to swimming performance. Following Brennen (20) and Namdeo et al. (22), one can estimate the total drag force on a flagellum, to leading order, as the linear summation of two parts: the drag of the flagellum without mastigonemes and the drag of each mastigoneme. Because the expected drag coefficients depend linearly on length and weakly on radius, hydrodynamic effects of the radius are neglected and only those of the total lengths are compared. The mastigonemes are relatively long (*l*_*m*_ ∼0.8 *μ*m) and are present at high densities (∼10 per *μ*m). Their total combined length is an order of magnitude longer than the flagellum (130 *μ*m compared to 12 *μ*m). Therefore, if they would not deflect, we expect free-swimming speed to be significantly affected by their presence (20, 21, 22).

### Observations of freely swimming cells

After confirmation of the lack of mastigonemes in *mstg* and the presence of mastigonemes in *cc125* and *cw15*, we compared the free-swimming speed, beating frequencies, and turning rates of the three strains. The algae were observed in flow chambers 20 mm wide and 2 mm in height. The observed cells swam 100 *μ*m above the closest wall to minimize any near-field wall effects.

Our observations were made using high-speed videography with spatial resolution of 0.1 *μ*m px^-1^. The videos were taken at a frame rate of 301 Hz, well above the minimum sampling rate required to resolve their flagella beating at frequencies $f_{0}\approx 50$ Hz. An image sequence from a typical observation is shown in Fig. 2 *a*. The average duration of each acquisition was 0.9 s.

The red curve in Fig. 2 *a* depicts the trajectory of the cell body’s centroid, and the inset shows a typical plot of the instantaneous swimming speed as a function of time. From the trajectory and instantaneous velocity, we extract the mean swimming speed, beating frequency $f_{0}$, and maximum swimming speed $U_{max}$. The white arrows in Fig. 2 *a* represent the heading direction *θ* of the swimming cell within the imaging plane, which is used to compute the turning rate.

The swimming trajectories of the cells were helical, as reported before (53,54). Our measurements were two-dimensional projections of this 3D motion, and this is the typical methodology for velocity measurements adopted in the literature (55). This approach and its reliability are detailed in the Supporting Materials and Methods.

Fig. 2 *b* shows the average swimming speed distributions for *mstg*, *cw15*, and *cc125* cells, from left to right, respectively. The distributions in mean speeds are not statistically different between the different cell strains (Kruskal-Wallis one-way analysis of variance (ANOVA), p>0.05). Our observations differ from those reported for cells with mastigonemes removed by an antibody in which a decrease of 20–30% was observed after their removal (35). The mean swimming speed of the wild-type cells measured by Nakamura et al. was 137.3 ± 16.0 *μ*m s^-1^ (35), whereas ours was 90.2 ± 39.0 *μ*m s^-1^ (average ± standard deviation). Although our observed swimming speeds are a bit lower, both observations agree with those previously reported for cells swimming in 3D (110±12 *μ*m s^-1^ (39), 136±12 *μ*m s^-1^ (56), and ∼130 *μ*m s^-1^ (57,58)).

The beating frequency $f_{0}$ distributions are represented in Fig. 2 *c*. We find that the cell-wall-deficient strains (both *mstg* and *cw15*) exhibit significantly higher beating frequencies ($f_{0}=57.0$ ± 2.9 and 55.2 ± 5.0 Hz, respectively) when compared to *cc125* cells ($f_{0}=52.2$ ± 4.1 Hz). The higher frequencies may be a result of the lack of cell wall. The two flagella protrude from the cell wall, and possibly make contact with it while beating. It is possible that either the mechanical confinement or coupling provided by the cell wall influences beating frequency because mechanical coupling between flagella is known to affect their synchronized beating frequency (39,59).

In Fig. 2 *d*, we present the distributions of the maximum speed $U_{max}$, scaled with the characteristic velocity $U_{0}=Lf_{0}$ to account for the variability in beating frequency and flagella length. The maximal speed $U_{max}$ was computed, for each track, as the median value of the peak velocities of each beat cycle (see *inset* of Fig. 2 *a*). We did not observe any significant differences in $U_{max}/U_{0}$ between the strains (Kruskal-Wallis one-way ANOVA, p>0.05). Therefore, even though the *mstg* and *cw15* strains beat faster than *cc125*, they were still propelling themselves at the same maximum speed during their power stroke relative to their flagella beating speed $U_{0}$. Previous observations of *U*_*max*_ ∼320 *μ*m s^-1^ (58) for *cc125* are within the range of our observations of $U_{max}=254.3$ ± 110.1 *μ*m s^-1^.

Incidentally, Fig. S1 shows the turning rate distributions for *mstg*, *cw15*, and *cc125* cells and an image sequence of a representative turning event with a large turning rate. The turning rates were determined from the time derivative of the heading direction *θ* or (dθ/dt). This derivative was computed for every time step, the absolute value of the derivative is taken, and then an average value for the duration of each acquisition was reported as the turning rate or $\bar{\left|d\theta /dt\right|}$. We found that *mstg* had a significantly higher turning rate compared to *cw15* and *cc125*. However, upon close inspection of the recordings, we see the increased turning to be a direct result of a decrease in symmetry between the beating of the two flagella, either because of slipping (Video S1), in which the flagella briefly lose their synchrony, or because of an asymmetry in the flagellar kinematics (Video S2), in which one flagellum swipes a larger area than the other.

Video S1. Free Swimming Cell Exhibiting Large Turning Rate Due to a Slip Event, where Flagella Lose Synchronous Beating This behavior does not produce the typical forward swimming behavior normally exhibited by the cells, such as in Fig. 2*a*, but instead results in the cell turning dramatically without forward progress. The cell turns completely around while staying in the same place. Video is slowed 30X.

Video S2. Free Swimming Cell Exhibiting Large Turning Rate Due to Asymmetry in Flagella Swiping Area, where One Flagellum Swipes a Larger Area than the Other Specifically, the flagellum on the left swipes a larger area than the one on the right. This results in a larger hydrodynamic thrust generated by the flagellum on the left, which causes the cell to turn to the right. Video is slowed 30X.

From our observations of freely swimming cells, we find that mastigonemes do not enhance swimming performance. We find no significant differences in either mean or maximum forward-swimming speeds between cells with or without mastigonemes. Our results differ from the observations of Nakamura et al. (35), which used a monoclonal antibody to remove the mastigonemes and observed a decrease of the swimming velocity for the cells without mastigonemes.

### Comparison of flagellar waveform

The swimming velocity of flagellated organisms is determined by both the hydrodynamic drag on the flagella and the kinematics of the flagellar deformation. Both need to be fully characterized to assess the hydrodynamic effect of mastigonemes.

**Figure 3:**
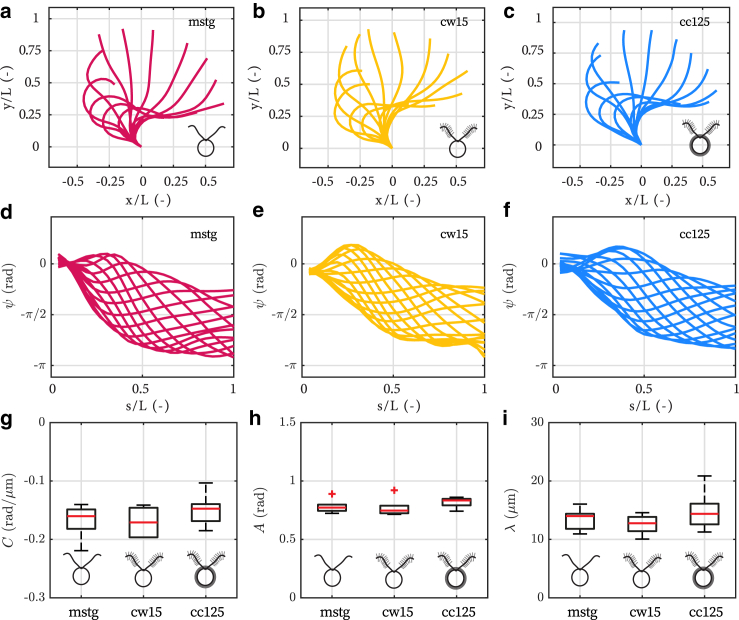
Flagellar gait kinematics. (*a*–*c*) Representative flagellar waveforms with respect to the cell's frame of reference (*x*, *y*), defined in Fig. 1*a*, for (*a*) *mstg*, (*b*) *cw15*, and (*c*) *cc125* cells. (*d*–*f*) Representative flagellar waveforms with respect to the local frame of reference (*s*, *ψ*), defined in Fig. 1*a*, for (*d*) *mstg*, (*e*) *cw15*, and (*f*) *cc125* cells. Here, *ψ* is the local tangent angle, and *s* is the arc length along the centerline of the flagellum. The flagellum length *L* is used to normalize spatial coordinates *x*, *y*, and *s*. (*g*–*i*) Comparison of fitting parameters for flagellar waveforms of *mstg*, *cw15*, and *cc125* cells from left to right, respectively. The parameters are (*g*) average curvature *C*, (*h*) traveling wave amplitude *A*, and (*i*) wavelength *λ*. The sample size for (*g*–*i*) is N=6 for each strain. To see this figure in color, go online.

Therefore, we proceed by analyzing the flagellar gait kinematics to determine whether strain mutations and their subsequent morphological differences affect beating patterns. High-speed videos of flagellar beating of cells held by a micropipette were recorded at 700 Hz. Typical flagellar waveforms are shown with respect to the cell’s frame of reference (*x*, *y*) in Fig. 3, *a*–*c*. The cell’s frame of reference is defined schematically in the inset of Fig. 1 *a*. To quantify gait kinematics, we parameterized the flagellar waveform of cells from each strain following the method used in Geyer et al. (60) and similar to VanderWaal et al. (61) and Bottier et al. (62).

This parameterization divides the motion of a flagellum into a static and dynamic component, where the flagellar motion is represented as a circular arc (static) and a sinusoidal traveling wave (dynamic). For the analysis, we measured the local angle *ψ* tangent to the flagellum for 26 discrete points along the flagellar arc length *s* for each recorded frame at time *t*, which resulted in a relationship for ψ(s,t). The parameters *s* and *ψ* are defined in Fig. 1 *a*. Typical flagellar waveforms are shown with respect to the local frame of reference (*s*, *ψ*) in Fig. 3, *d*–*f*. The static component of the motion is $\psi_{1}\left(s\right)=Cs$, where *C* represents the mean curvature of the flagellum. The dynamic component of the motion is $\psi_{2}\left(s,t\right)=A\text{sin}\left(2\pi \left(f_{0}t+s/\lambda \right)\right)$, where *A* is the amplitude of the traveling wave, $f_{0}$ is the beating frequency, and *λ* is the wavelength.

Combining the static $\psi_{1}\left(s\right)$ and dynamic $\psi_{2}\left(s,t\right)$ components yields an expression for the tangent angle along the flagellar length, or $\psi \left(s,t\right)=Cs+A\text{sin}\left(2\pi \left(f_{0}t+s/\lambda \right)\right)$. The parameters *C*, *A*, and *λ* were extracted for each flagellum of N=6 cells per strain. For all three strains, we compared the mean curvature *C* (Fig. 3 *g*), traveling wave amplitude *A* (Fig. 3 *h*), and wavelength *λ* (Fig. 3 *i*). In each panel, *mstg*, *cw15*, and *cc125* are displayed respectively from left to right. For all three waveform parameters, we find no statistical differences between the strains (Kruskal-Wallis one-way ANOVA, p>0.05).

The mean curvature is C=−0.17±0.03, −0.17±0.02, and −0.15±0.02 rad *μ*m^-1^ (average ± standard deviation) for *mstg*, *cw15*, and *cc125* cells, respectively. The values of *C* for *cc125* cells are in agreement with those of previous studies (−0.17±0.005 rad *μ*m^-1^ (60,63) and −0.21±0.07 rad *μ*m^-1^ (62)). The traveling wave amplitude is A=0.78±0.05, 0.77±0.07, and 0.82±0.04 rad (average ± standard deviation) for *mstg*, *cw15*, and *cc125* cells, respectively. The values of *A* for *cc125* are also in agreement with those of previous studies (1.08±0.09 rad (60,63) and ∼0.89 ± 0.06 rad (62)). Finally, the wavelength is λ=13.5±1.7, 12.6±1.5, and 14.9±3.1 *μ*m (average ± standard deviation) for *mstg*, *cw15*, and *cc125* cells, respectively. Values of *λ* for *cc125* cells are again in agreement with previous studies (15.1±2.0 *μ*m (60,63)).

In conclusion, because there are no significant differences for all three parameters that characterize the flagellar waveform, the flagellar gait kinematics and deformations appear unaffected by the presence of mastigonemes.

### Optical tweezers-based velocimetry

After having observed no significant differences in the free-swimming characteristics or flagellar kinematics of cells with and without mastigonemes, we directly probed the hydrodynamics of captured cells using OTV (33). This technique allowed us to locally measure the flow field generated by beating flagella with and without mastigonemes.

A schematic depicting our OTV experiments is shown in Fig. 4 *a*, with a picture from our experiments in the inset at the top right. A polystyrene bead of diameter 2 or 5 *μ*m was trapped in the vicinity of a flagellum. We measured the local two-dimensional flow field u(t)=(u,v), *u* (axial) and *v* (lateral), generated by the cell at the location of the bead. The flow field was recorded at increasing lateral distances from the cell by moving the bead along the *y*-axis. This procedure was previously implemented to study the flow around *cc125* cells (33). We also defined the flagellar phase *ϕ* to represent the flagellar shapes during a beat cycle. The most forward-reaching shape, as shown in the inset of Fig. 4 *a*, was set as the beginning of a cycle (ϕ=0). Typically, the power stroke ends at ϕ≈π, shown in Fig. 4 *a*.

The periodic signal of axial *u* and lateral *v* velocities were recorded over successive flagellar beat cycles with a high temporal resolution of 10 kHz; see Fig. 4, *b* and *c*. To compare the hydrodynamics of individual cells across strains, we reconstructed an average beat cycle for each cell and extracted the amplitude of the oscillations and the time-averaged flow velocity generated in the axial (Δu' and $\bar{u}$, respectively) and lateral directions (Δv' and $\bar{v}$, respectively) for each individual cell.

Fig. 4, *d* and *e* represent the flow velocity of a typical beat cycle, constructed as the median over 50 cycles. The asymmetry in the power-recovery stroke is clearly visible in the axial flow *u*, with the power stroke generating a strong positive flow for the first half of the cycle (ϕ<π), and the recovery stroke subsequently generating a weaker negative flow. The flow amplitudes (Δu' and Δv') and average flows ($\bar{u}$ and $\bar{v}$) are extracted from the typical beat cycle as represented on Fig. 4, *d* and *e*. Schematics representing the shapes at each flagellar phase *ϕ* are displayed in the upper horizontal axis of Fig. 4 *d*.

**Figure 5:**
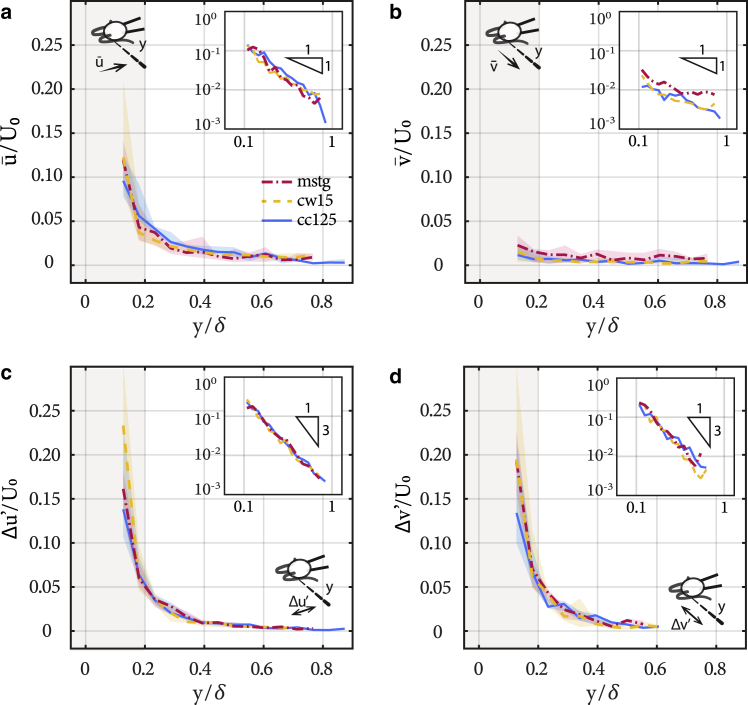
Hydrodynamics of beating flagella from optical tweezers-based velocimetry (OTV). (*a* and *b*) Relationship between average (*a*) axial $\bar{u}$ and (*b*) lateral $\bar{v}$ flows and distance *y* from the midplane of the cell. (*c* and *d*) Relationship between flow amplitudes in (*c*) axial *Δu*′ and (*d*) lateral *Δv*′ directions and distance *y*. The red dash-dot line is for *mstg*(N=9), the yellow dashed line is for *cw15*(N=11), and the solid blue line is for *cc125*(N=14) cells. The lines represent the median, whereas the shaded region represents the interquartile. The distance *y* is normalized by $\delta =\sqrt{\nu /f_{0}}$, where *ν* is the kinematic viscosity of water. Insets show data plotted in log-log scale to highlight the rates of spatial decay for the flows. To see this figure in color, go online.

We use the flow parameters $\bar{u}$, $\bar{v}$, Δu', and Δv' to investigate the flow field around beating cells and compare between the three strains to determine the hydrodynamic effects of mastigonemes. The distributions of average flows and amplitude of flow oscillations are reported in Fig. 5. The distance *y* from the cell is scaled by the diffusive length scale $\delta =\sqrt{\nu /f_{0}}$, where *ν* is the kinematic viscosity of water. The insets present the same data in log-log scale and highlight the rate of spatial decay of the velocity field. The beating flagella generate a flow, which is significantly stronger in the axial *x*-direction Fig. 5 *a* compared with the lateral *y*-direction Fig. 5 *b*. The magnitude of the average flow decays in ∼1/*y*, as expected from the stokeslet flow (Fig. 5, *a* and *b*). The amplitude of the flow oscillations Δu' and Δv' is large in the vicinity of the flagella but decays in ∼1/*y*^3^, faster than the average flow (33); see Fig. 5, *c* and *d*.

The spatial distributions of average velocity and amplitude of oscillation reveal no significant differences in the flow fields generated by the three different strains; see Fig. 5. The measurements were performed for each strain for different cells with N=9−14. The variability of the measurements for a given strain is represented by the interquartile range in Fig. 5 and is comparable to the variability observed in swimming velocities; see Fig. 2. The differences in the flow fields generated by *mstg*, *cw15*, and *cc125* are within the variability observed within a given strain. Overall, experimental measurements do not evidence an increase in flow generation because of the presence of mastigonemes.

### Comparisons with hydrodynamic modeling

To directly quantify the hydrodynamic effect of mastigonemes, we performed computational fluid dynamic simulations to compute the flow field close to the flagella and directly compared them with our time-resolved flow measurements. To account for individual cell to cell variations between our experiments, we first tracked the flagellar shapes for each recording (see Fig. 3) and used the deformations as boundary conditions for the simulation. The flagellar kinematics are tracked using high-speed videography (∼700 Hz). The flow is simulated by solving Stokes equations, which have been shown previously to accurately model fluid flows in the near-field, for y<0.2δ (33). Our hydrodynamic model assumes the flagella to be cylindrical, and so, without mastigonemes; therefore, agreement with measurements from *mstg* and disagreement with measurements from *cw15* and *cc125* would reveal the hydrodynamic contributions from mastigonemes.

**Figure 6:**
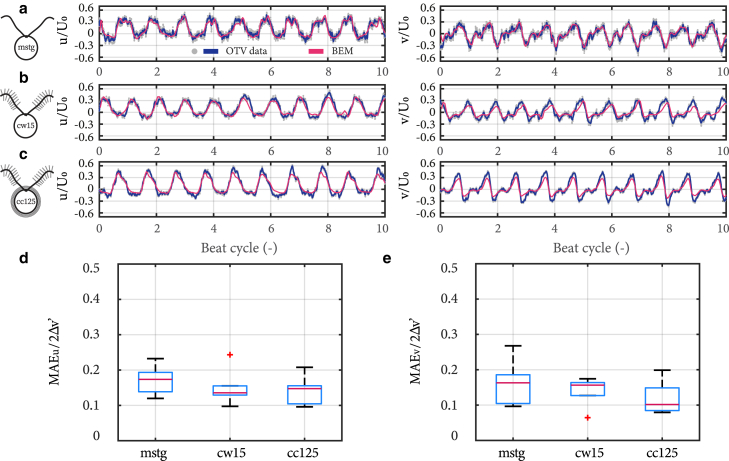
Near-field hydrodynamics of beating flagella. (*a*–*c*) Axial *u* (*left*) and lateral *v* (*right*) flow as a function of beat cycle for a typical (*a*) *mstg*, (*b*) *cw15*, and (*c*) *cc125* cell. The red solid curve is the prediction from hydrodynamic modeling (BEM). (*d* and *e*) Comparison of MAE of BEM predictions for (*d*) axial flows MAE_*u*_ and (*e*) lateral flows MAE_*v*_, for *mstg*, *cw15*, and *cc125* strains from left to right, respectively. The data represented are from within ∼20 *μ*m of a beating cell (y<0.2δ), or the lightly shaded areas in Fig. 5. The sample size for (*d*) and (*e*) is N=6 for each strain. To see this figure in color, go online.

Fig. 6, *a*–*c* show the relationship between flow velocity, *u* (left) and *v* (right), and beat cycle for (Fig. 6 *a*) *mstg*, (Fig. 6 *b*) *cw15*, and (Fig. 6 *c*) *cc125* cells. The red curves represent our simulations using a hybrid BEM and slender-body approach to solve Stokes equations around beating cells (33,45,51,52). Video S3 shows a representative flagellar stroke from experiments, followed by the flow field predicted from the simulations.

Video S3. One Beating Cycle of a Captured Cell from OTV Experiments, Followed by Flow Field Generated from Numerical Simulation Video is slowed 120X.

Because our simulations assume that the flagella do not have mastigonemes, we begin by comparing with experiments for *mstg*. From Fig. 6 *a*, we observe good agreement between measurements and BEM. Quantitatively, we compute the mean absolute error (MAE), which is the average deviation of the instantaneous flow velocity between BEM and experiments, or${\mathrm{MAE}}_{u}=\sum_{i=1}^{n}\left|u_{s,i}-u_{i}\right|/n$, where $u_{s,i}$ is the instantaneous velocity predicted by simulation and *n* is the total number of instantaneous points measured/simulated. In Fig. 6, *d* and *e*, we show the error of our simulations in the axial and lateral direction for *mstg* normalized by the flow amplitude, or (Fig. 6 *d*) ${\text{MAE}}_{\text{u}}/2\Delta u'$ and (Fig. 6 *e*) ${\text{MAE}}_{v}/2\Delta v'$, respectively. The error values are around 0.15. Therefore, our simulations accurately capture the flow field generated by beating flagella.

After validation of our simulations with the mastigonemeless mutant *mstg*, we proceed by comparing simulations and experiments for *cw15* and *cc125* cells. Similar to *mstg*, we find good agreement between experiments and BEM in Fig. 6, *b* and *c*, which are confirmed quantitatively in Fig. 6, *d* and *e*. Moreover, we find no statistical differences between errors in simulation predictions across the three strains (Kruskal-Wallis one-way ANOVA, p>0.05).

If the mastigonemes contributed to the hydrodynamics, we would have expected better agreement between measurements and simulations for the *mstg* cells because our modeled flagella are smooth. Instead, we find no differences between strains, and, in all cases, the observed hydrodynamics around beating cells are well captured by representing the flagella as smooth, i.e., neglecting the mastigonemes.

This is unlike what we expect if mastigonemes are rigid and dense and increase the effective width of a flagellum. By direct computation using BEM, we solve Stokes equations around slender flagella of different cross-sectional geometries and obtain their drag coefficients, see Supporting Materials and Methods for details. Assuming the mastigonemes do not deform, we estimate the drag coefficient of a straight flagellum of length L=12 *μ*m, width $w=2\left(r+l_{m}\right)=2$ *μ*m, and thickness h=2r. The width *w* accounts for the two rows of mastigonemes of length $l_{m}$ observed in Fig. 1 *b*. The geometry is shown in Fig. S2. We compare this value with the drag coefficient of a straight and smooth flagellum of length L=12 *μ*m and diameter 2r=0.5 *μ*m.

We find the drag increase is 50% for flow in the direction perpendicular to the mastigonemes, and 30% for flow in the direction parallel to them. The 50% increase is comparable with estimates of the drag coefficient around a cylinder of increased diameter from 2r=0.5 *μ*m to $2\left(r+l_{m}\right)=2$ *μ*m (64). Alternatively, for an undulating swimmer without a separate cell body (*C. elegans*), a 30% reduction in swimming speed is predicted for the same increase in diameter (24). Therefore, according to both models, the hydrodynamic effect of straight and rigid mastigonemes would be significant and clearly measurable given the accuracy of the OTV technique.

Overall, from our OTV observations and hydrodynamics modeling, we conclude that the mastigonemes do not contribute to the flows generated by beating flagella and do not have a direct hydrodynamic effect. There are no differences between the axial *u* or lateral flows *v* generated per stroke by *mstg*, *cw15*, or *cc125* cells. Additionally, our hydrodynamic model, which assumes smooth flagella without mastigonemes, accurately represents the fluid dynamics around both cells with mastigonemes (*cc125* and *cw15*) and cells without (*mstg*).

Since we do not observe any hydrodynamic differences because of the presence of mastigonemes, we can conclude that the increased turning rate in *mstg* is not due to a direct hydrodynamic effect of the mastigonemes and, more likely, results from either differences in flagella swiping areas or slipping events (see Observations of freely swimming cells). These behavioral differences would require further studies to determine whether and how they are linked to the presence of mastigonemes.

Our results refute the hypothesis currently accepted in the literature that the fibrous mastigonemes increase the effective area of *C. reinhardtii*’s flagella during their beating strokes (18). A possible explanation may be the flexibility of the mastigonemes of *C. reinhardtii*. Because they are thin and of the fibrous type (18), they may bend and deflect when the flagella moves through the fluid. As pointed out by Brennen (20) and Namdeo et al. (22), if the flexibility of the mastigonemes is below a critical value, then they would not contribute to the drag of the flagellum, and the drag force would be equivalent to that of a smooth flagellum.

## Conclusions

Our study thoroughly investigated the possible hydrodynamic contribution of the mastigonemes of *C. reinhardtii* using a mutant lacking these structures. In conclusion, we confirmed through TEM imaging that the *mstg* mutant used in our study lacks mastigonemes, whereas the *cw15* and *cc125* cells possess them. From observations of freely swimming cells, we find that mastigonemes do not increase swimming velocity. Furthermore, mastigonemes do not affect flagellar gait kinematics. Finally, from our OTV measurements, we find that mastigonemes do not help produce larger hydrodynamic forces or propulsive flows per stroke, as has been previously hypothesized (18,34,35). Future studies into the bending stiffness of *C. reinhardtii* mastigonemes may help confirm whether mastigonemes bend and deflect during swimming, which render them useless for hydrodynamic enhancement. Additionally, future studies into the effects of mastigonemes on swimming behavior could shed light on functions of mastigonemes other than mere hydrodynamics. Overall, our study found that mastigonemes do not contribute to the swimming propulsion of *C. reinhardtii*. Their function still remains enigmatic. More studies into their contributions to sensing, adhesion, or feeding may reveal their function and evolution.

## Author Contributions

D.W. and G.J.A. performed research. All authors designed research, analyzed data, and wrote the manuscript. D.T. and M.-E.A.-T. supervised the work and are both corresponding authors.

## Supporting Material

Document S1. Supporting Materials and Methods and Figs. S1 and S2

Document S2. Article plus Supporting Material
