# Supplementary material for: Fibrous Flagellar Hairs of Chlamydomonas reinhardtii Do Not Enhance Swimming
Source: Biophys J. 2020 May 19;118(12):2914–25. doi: 10.1016/j.bpj.2020.05.003 (PMC7300311; doi:10.1016/j.bpj.2020.05.003)
Supplement: Document S1. Supporting Materials and Methods and Figs. S1 and S2 [file mmc1.pdf]

Biophysical Journal, Volume *118*

Supplemental Information

Fibrous Flagellar Hairs of *Chlamydomonas reinhardtii* Do Not Enhance Swimming

Guillermo J. Amador, Da Wei, Daniel Tam, and Marie-Eve Aubin-Tam

Supplementary Information:
Fibrous flagellar hairs of *Chlamydomonas reinhardtii* do not enhance swimming

G.J. Amador, D. Wei, D. Tam, M.E. Aubin-Tam

1 Free swimming helical trajectory

For our measurements, we observed the 2D projections of the cells' 3D helical trajectories. As such, we may underestimate the trajectory length.

Here we use the parametric equations for a 3D helix with x the translational direction, to estimate the error in 2D measurements:

$$\begin{aligned} x(t') &= b\cos(t'), \\ y(t') &= ct', \\ z(t') &= b\sin(t'), \end{aligned} \tag{1}$$

Here, t' ranges from 0 to 2π for a single cycle, b is the radius of the helix, and c represents its wavelength. Following previous measurements [1, 2], we assume $b = 5$ μm. The wavelength c is found using the trajectory lengths we measured for the 2D projections assuming a helix frequency of 2 Hz [1, 2]. We calculate the 2D trajectory length L_{2D} as follows:

$$L_{2D} = \int_0^{2\pi} \sqrt{\left(\frac{dx}{dt'}\right)^2 + \left(\frac{dy}{dt'}\right)^2} dt', \tag{2}$$

which we approximate as the average swimming speed divided by the helix frequency, or $L_{2D} = 45$ μm. Substituting Eq. (1) into Eq. (2), we can solve for $c = 6.3$ μm. Using this value for c, we can find the 3D trajectory length L_{3D} following:

$$L_{3D} = \int_0^{2\pi} \sqrt{\left(\frac{dx}{dt'}\right)^2 + \left(\frac{dy}{dt'}\right)^2 + \left(\frac{dz}{dt'}\right)^2} dt'. \tag{3}$$

We can then estimate the error as $(L_{3D} - L_{2D})/L_{3D}$. Substituting Eq. (1) into Eq. (3), we get an error estimate of 10%.

2 Computation of hydrodynamic drag

To estimate the potential direct hydrodynamic effect of mastigonemes when fluid does not flow between the fibers, we compute the drag coefficients on slender flagella of different cross-sectional geometry using the boundary element method (BEM). Our numerical approach solves the Stokes equations using the completed double-layer boundary integral equation [3], with a completion flow due to a distribution of singularities along the central axis [4].

We estimate the drag coefficient on a straight flagellum without mastigonemes, which we represent as a straight cylindrical rod of length $L = 12\mu$m and radius $r = 0.25$ μm, see **Fig. S2**. The value obtained differs less than 1% from that predicted by the analytical solution $4\pi\mu L/(\ln(L/r) - 0.5 + \ln 2)$, derived by Cox [5] for a motion in the direction perpendicular to the centerline, corresponding to a motion in the Y or Z direction on **Fig. S2**.

This value is compared with a model flagellum with straight rigid mastigonemes, which we represent as a rigid flagellum with an increased effective area. This flagellum is represented as a flattened flagellum of length $L = 12\mu$m, with the same thickness as the cylindrical flagellum $2r = 0.5\mu$m but with a width corresponding to that of the axoneme increased by the two rows of mastigonemes $2(r + l_m) = 2$ μm, see **Fig. S2**. We find the drag coefficient to be significantly larger than for the cylindrical flagellum, with an increase in drag of 32% for a motion in the Y-direction and 50% for a motion in the Z-direction.

3 Supplementary movie captions

Movie S1. Free swimming cell exhibiting large turning rate due to a slip event, where flagella lose synchronous beating. This behavior does not produce the typical forward swimming behavior normally exhibited by the cells, such as in **Fig.2**a, but instead results in the cell turning dramatically without forward progress. The cell turns completely around while staying in the same place. Video is slowed 30X.

Movie S2. Free swimming cell exhibiting large turning rate due to asymmetry in flagella swiping area, where one flagellum swipes a larger area than the other. Specifically, the flagellum on the left swipes a larger area than the one on the right. This results in a larger hydrodynamic thrust generated by the flagellum on the left, which causes the cell to turn to the right. Video is slowed 30X.

Movie S3. One beating cycle of a captured cell from optical tweezers-based velocimetry (OTV) experiments, followed by flow field generated from numerical simulation. Video is slowed 120X.

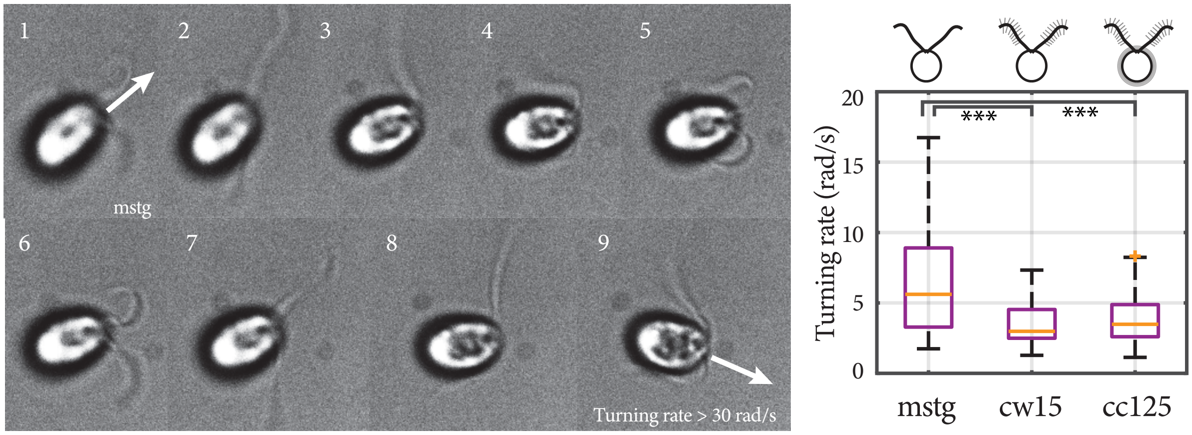

Figure S1: Turning in free swimming observations. (1-9) Image sequence of *mstg* cell during a turning event with a large turning rate. The white arrows represent the cell's heading direction. The time step between the images is 3.3 ms. Comparison of average turning rate for *mstg*, *cw15*, and *cc125* cells from left to right, respectively. Here, $***$ represents $p < 0.001$ following a Kruskal-Wallis One-Way ANOVA statistical test.

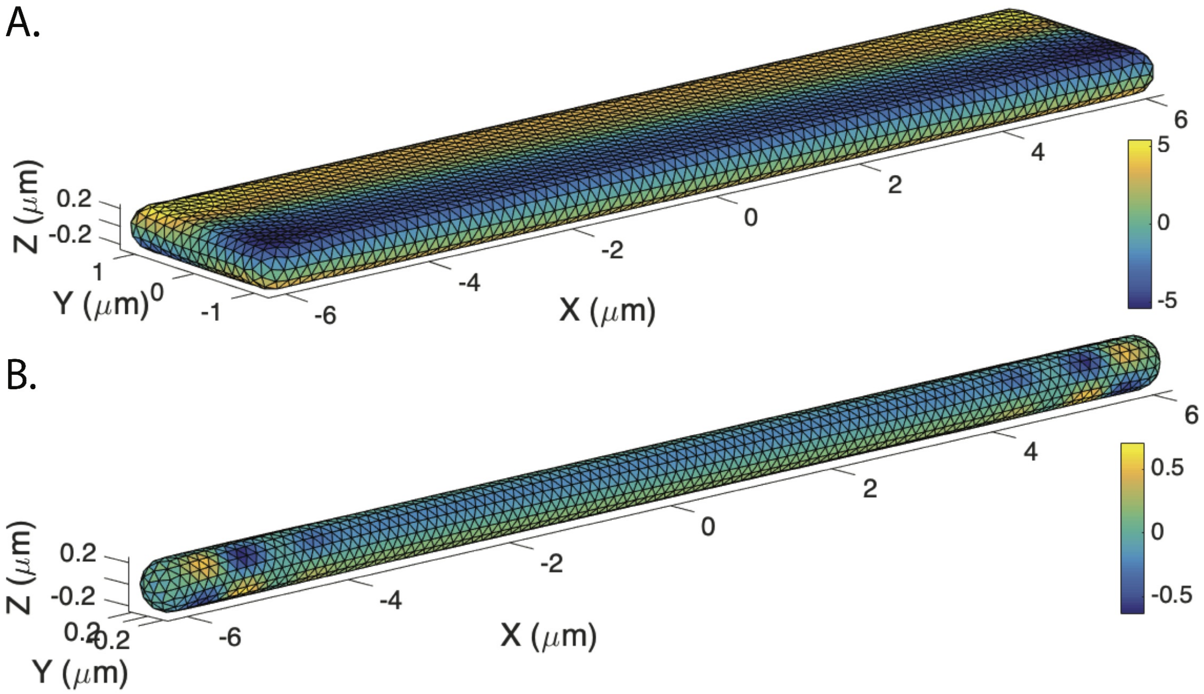

Figure S2: Drag coefficient for different cross-sections. Computation of drag coefficient using BEM on an unstructured triangular mesh. The colormap represents the intensity of one component of the double layer potential. (a) Model flagellum representing a flagellum with two rows of rigid mastigonemes where fluid does not flow between them. (b) Cylindrical flagellum without mastigonemes.

References

[1] Isogai, N., Kamiya, R. & Yoshimura, K. Dominance between the two flagella during phototactic turning in *Chlamydomonas*. *Zoological Science* **17**, 1261–1266 (2000).

[2] Crenshaw, H. C. A new look at locomotion in microorganisms: Rotating and translating. *American Zoologist* **36**, 608–618 (2015).

[3] Power, H. & Miranda, G. Second kind integral equation formulation of Stokes flows past a particle of arbitrary shape. *SIAM Journal on Applied Mathematics* **47**, 689–698 (1987).

[4] Keaveny, E. E. & Shelley, M. J. Applying a second-kind boundary integral equation for surface tractions in stokes flow. *Journal of Computational Physics* **230**, 2141–2159 (2011).

[5] Cox, R. The motion of long slender bodies in a viscous fluid Part 1. General theory. *Journal of Fluid Mechanics* **44**, 791–810 (1970).
