# Supplementary material for: Fibrous Flagellar Hairs of Chlamydomonas reinhardtii Do Not Enhance Swimming
Source: Biophys J. 2020 May 19;118(12):2914–25. doi: 10.1016/j.bpj.2020.05.003 (PMC7300311; doi:10.1016/j.bpj.2020.05.003)
Supplement: Document S2. Article plus Supporting Material [file mmc5.pdf]

Biophysical Journal
Article

Biophysical Society

Fibrous Flagellar Hairs of *Chlamydomonas reinhardtii* Do Not Enhance Swimming

Guillermo J. Amador,[1,2] Da Wei,[2] Daniel Tam,[1,*] and Marie-Eve Aubin-Tam[2,*]
[1]Laboratory for Aero and Hydrodynamics and [2]Department of Bionanoscience, Kavli Institute of Nanoscience, Delft University of Technology, Delft, the Netherlands

ABSTRACT The flagella of *Chlamydomonas reinhardtii* possess fibrous ultrastructures of a nanometer-scale thickness known as mastigonemes. These structures have been widely hypothesized to enhance flagellar thrust; however, detailed hydrodynamic analysis supporting this claim is lacking. In this study, we present a comprehensive investigation into the hydrodynamic effects of mastigonemes using a genetically modified mutant lacking the fibrous structures. Through high-speed observations of freely swimming cells, we found the average and maximum swimming speeds to be unaffected by the presence of mastigonemes. In addition to swimming speeds, no significant difference was found for flagellar gait kinematics. After our observations of swimming kinematics, we present direct measurements of the hydrodynamic forces generated by flagella with and without mastigonemes. These measurements were conducted using optical tweezers, which enabled high temporal and spatial resolution of hydrodynamic forces. Through our measurements, we found no significant difference in propulsive flows due to the presence of mastigonemes. Direct comparison between measurements and fluid mechanical modeling revealed that swimming hydrodynamics were accurately captured without including mastigonemes on the modeled swimmer's flagella. Therefore, mastigonemes do not appear to increase the flagella's effective area while swimming, as previously thought. Our results refute the longstanding claim that mastigonemes enhance flagellar thrust in *C. reinhardtii*, and so, their function still remains enigmatic.

SIGNIFICANCE Eukaryotic flagella are generally modeled as cylindrical and smooth, but surface structures are common and can significantly affect motility. *Chlamydomonas reinhardtii* has long served as the model organism for studies on the structure, assembly, and function of eukaryotic flagella. Decades ago, their flagella were observed to possess nanometer-thick fibers known as mastigonemes or flagellar hairs. Based on evidence from another alga *Ochromonas*, these structures have been widely hypothesized to increase the effective area of flagella and enhance thrust during swimming. However, in this study, we refute this hypothesis. Specifically, we show that they do not increase swimming speed, alter flagellar deformations, or increase hydrodynamic thrust. Therefore, their function remains enigmatic but seemingly important, considering they are actively replaced after removal.

INTRODUCTION

Hair-like, or fibrous, structures are ubiquitous features in biology. They are present in organisms ranging from plants to mammals, birds, and insects. Their functions range from temperature control (1) to sensing (2), cleaning (3), adhering (4), flying (5,6), and feeding (7,8). A number of these functions rely on hydrodynamic interactions between the fibrous structures and the surrounding fluid. The structures provide a resistance to fluid flow, and this resistance has been exploited by various organisms, e.g., to enhance the spatial resolution of chemoreception in moth antennae (9), to minimize evaporation from and deposition to mammalian (10) and insect (11) eyes, to capture food particles in aquatic filter feeders (7), to entrain viscous nectar in pollinators (8), and to generate aerodynamic lift in flapping flight of birds (12) and insects (6). Fluid-structure interactions thus appear to play a crucial role in the specialized function of certain fibrous structures.

In addition to multicellular organisms, single-celled eukaryotes have been observed to possess nanometer-scale hair-like fibers, or mastigonemes (13–17). Mastigonemes are present on the flagella, and so are also referred to as flagellar hairs (18). Because these organisms swim via drag-based interactions between their solid flagella and

Submitted November 25, 2019, and accepted for publication April 27, 2020.
*Correspondence: d.s.w.tam@tudelft.nl or m.e.aubin-tam@tudelft.nl
Guillermo J. Amador and Da Wei contributed equally to this work.
Editor: Kinneret Keren.
https://doi.org/10.1016/j.bpj.2020.05.003
© 2020 Biophysical Society.
This is an open access article under the CC BY license (http://creativecommons.org/licenses/by/4.0/).

fluid-filled environments using wavelike stroke patterns (19), their mastigonemes are expected to affect swimming performance through hydrodynamic interactions.

Previous studies have shown significant contributions to drag from rigid mastigonemes on the anterior flagellum of the golden algae *Ochromonas* (20–22). These algae swim using a traveling wave through their anterior flagellum. Using both theoretical (20) and numerical (22) modeling, these studies determined that mastigonemes with a particular stiffness are necessary to capture the swimming speed and direction observed experimentally (21). If the mastigonemes are too flexible, then the models predict they would bend and their contribution to the overall drag of the flagellum would be negligible; however, above a certain stiffness, they contribute significantly. In fact, in these swimmers, the hydrodynamic effect of mastigonemes is so significant that swimming direction is contrary to the expected direction for a flagellum without mastigonemes (19). A similar thrust reversal was observed in artificial helical microswimmers with rigid mastigoneme-like structures (23).

Microscopic swimmers vary significantly in morphology and swimming gaits. In swimmers in which the cell body and undulating filament are one and the same, such as *Caenorhabditis elegans*, an increase in thickness would result in a significant decrease in swimming speed (24). However, when an undulating flagellum is pushing a cell body, a thicker flagellum can lead to an increase in swimming speed (25). Besides undulating a posterior flagellum, another common swimming strategy observed in eukaryotic cells is the use of two anterior flagella. These two flagella beat in a synchronous pattern akin to a human swimmer's breaststroke. Such a swimming gait is exhibited by the organism used in this study, *Chlamydomonas reinhardtii* (Fig. 1 *a*). The flagella of *C. reinhardtii* cells were inherited from the common ancestor of land plants and animals; as such, this organism has long served as the model organism used for studies of the structure, assembly, and function of eukaryotic flagella (26).

The mastigonemes of *C. reinhardtii* are of the fibrous type, as opposed to the tubular mastigonemes found in other algae (18) such as *Ochromonas*. Whereas tubular mastigonemes are thicker and their hydrodynamics have been studied extensively (20,21), studies into the hydrodynamics of fibrous mastigonemes are lacking, despite the fact that their bearer, *C. reinhardtii*, has drawn much attention and its swimming kinematics are well characterized (27–33).

Following their initial observation and characterization (15), the mastigonemes of *C. reinhardtii* (shown in Fig. 1 *b*) were widely hypothesized to enhance hydrodynamic performance of flagella by increasing the flagella's effective area (18,34) because of their long length and high density. However, experimental evidence supporting their hydrodynamic function has been limited to the observation of a decrease in swimming speed of 20–30% for *C. reinhardtii* cells treated with mAb-MAST1 (35), a monoclonal antibody that was reported to trigger the loss of mastigonemes. Because this is the only experimental observation on the hydrodynamics of fibrous mastigonemes, and other information such as their effects on gait kinematics and drag force are lacking, we were motivated to gather more detailed knowledge in this regard. We thus conducted our research to resolve the function of these ultrastructures at the single-cell level.

In this study, we used a genetically modified mutant that possesses no mastigonemes (36), and therefore avoid using an antibody to remove mastigonemes. This mutant, referred to as *mstg*, is generated via insertional mutagenesis in which the mutation is inserted into the gene predicted to code for a mastigoneme protein (GenBank: AF508983, MST1) (37). The parent strain used for mutation is *cw15*,

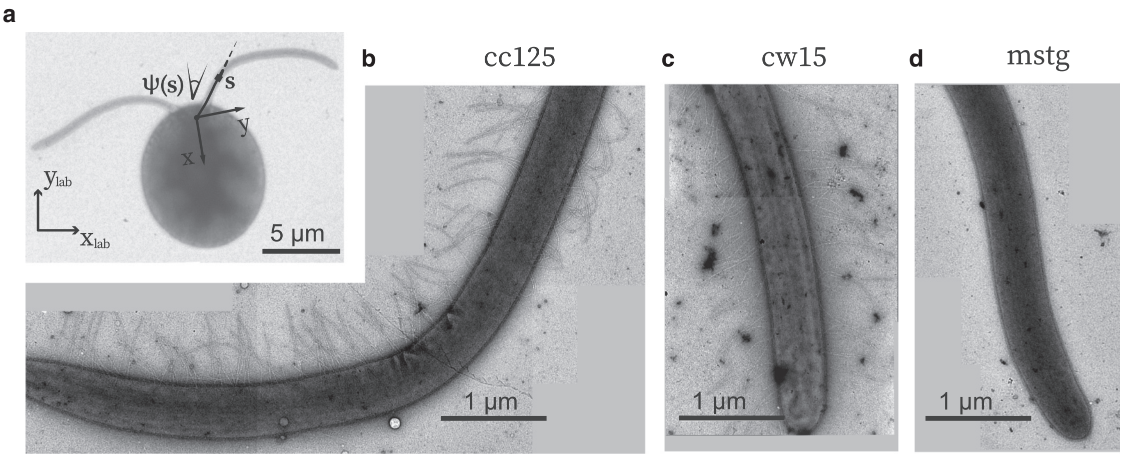

FIGURE 1 Microscopy of flagella with and without mastigonemes. (*a*) TEM image of wild-type (*cc125*) *C. reinhardtii*. (*b–d*) Representative TEM images of flagella from (*b*) *cc125*, (*c*) cell-wall-deficient strain (*cw15*), and (*d*) cell-wall-deficient strain without mastigonemes (*mstg*). The *cc125* and *cw15* cells clearly exhibit mastigonemes, whereas the *mstg* cells do not.

a cell-wall-deficient strain of the wild-type *C. reinhardtii*. Although *cw15* lacks a cell wall (38), it still possesses mastigonemes on its flagella. For completeness, we also included the extensively characterized wild-type strain cc-125 mt + as another control group and denote it as *cc125*. Through a thorough comparative study in the cells' free-swimming dynamics, flagellar kinematics, and flagellar hydrodynamics across the three strains (*mstg*, *cw15*, and *cc125*), we investigate the potential hydrodynamic effects of mastigonemes.

We first assessed the presence of mastigonemes in the flagella of the three strains of *C. reinhardtii* with transmission electron microscopy (TEM). Through these observations, we confirmed the presence of mastigonemes in *cc125* and *cw15*, and their absence in the *mstg* mutant. We then analyzed the trajectories of freely swimming cells. In these observations we compared the swimming speeds, beating frequencies, and turning rates to determine how mastigonemes affect locomotory performance and kinematics. Then, we compared the flagellar gait kinematics with high-speed videography of captured single cells to determine how they are affected by the removal of the cell wall or mastigonemes. Next, we performed velocimetry measurements using optical-tweezers-based flow velocimetry (OTV), following the protocol in (33). The OTV technique measures the displacement of an optically trapped particle within the laser, from which fluid force and velocity are inferred directly. Our technique provides high temporal resolution to characterize the hydrodynamic effects of the mastigonemes near a captured algal cell. Finally, following our measurements, we compared our experimental results to numerical simulations that solve Stokes equations around a beating cell assuming smooth (hairless) flagella.

MATERIALS AND METHODS

Cells and cell culture

Three strains of *C. reinhardtii* were used in experiments: wild-type *cc125* (cc-125 mt+), cell-wall-deficient *cw15* (cc-4453 mt−), and cell-wall- and mastigoneme-deficient *mstg*. The *mstg* mutant, LMJ.RY0402.136134, was generated by the Chlamydomonas Library Project using *cw15* as the parent strain (36). The *cw15* and *mstg* strains obtained from the Chlamydomonas Resource Center were propagated in the laboratory conditions over several months for acclimation to temperature and humidity.

All three strains were grown in identical conditions following established protocols, see (39). Specifically, they were cultured in Tris-minimal medium (pH 7) with sterile air bubbling. The cultures were subjected to light/dark (14:10 h) cycles with light intensity of 230 μE m^{-2} s^{-1}. They were harvested on the fourth day after inoculating the liquid culture when it reached a density of $\sim 2 \times 10^5$ cells mL^{-1} and then diluted in fresh Tris-minimal medium (pH 7).

Transmission electron microscopy of flagella and mastigonemes

TEM observations were done with a JEM-1400 (JEOL, Tokyo, Japan). Liquid cultures of *cc125*, *cw15*, and *mstg* were simultaneously harvested and washed with fresh Tris-minimal medium. Thereafter, we centrifuged the cells (600 × *g* for 5 min) twice to increase the cell concentration.

Immediately after the concentrated cell suspensions were obtained, a droplet was placed on a carbon-coated copper TEM grid for a duration of 2 min. Afterwards, the excess cell suspension was removed by blotting with a filter paper, and the grids were stained by immersion in 2% uranium acetate during 1 min. We only recorded the cells with clearly attached flagella and with no sign of cell lysis.

High-speed imaging and light microscopy

For our observations of freely swimming and captured cells, we used an inverted microscope (Nikon Eclipse Ti-U; Nikon, Tokyo, Japan) with a 60× water immersion objective (Nikon CFI Plan Apo VC 60× NA = 1.20; Nikon). The high-speed videography was conducted using a sCMOS high-speed camera (PCO.edge; PCO Tech, Kelheim, Germany). We obtained a spatial resolution of 0.1 μm px^{-1}and temporal resolution of 301 and 699 Hz for observations of freely swimming and captured cells, respectively.

All cells were observed in conditions similar to those in Wei et al. (33) and Quaranta et al. (39). Cells suspended in Tris-minimal medium were placed within a custom flow chamber (20 mm wide by 2 mm high). The chamber was sealed using silicone oil to ensure no background flows were present due to fluid evaporation. Before every experiment, suspended particles or cell debris were observed to confirm the absence of background flows. The flow chamber was fixed onto a piezoelectric stage (Nano-Drive; Mad City Labs, Madison, WI) integrated on the inverted microscope. For the free-swimming experiments, the cells were then observed directly using high-speed videography.

For the flagellar kinematics and OTV experiments, the cells were held by micropipettes made of borosilicate glass. The micropipette was mounted onto a micromanipulator (SYS-HS6; World Precision Instruments, Sarasota, FL).

Analysis of freely swimming cells

Frame rates of the videography for free-swimming observations were set at 301 Hz, which was sufficient to detect the cells' motion and resolve their flagella beating at $f_0 \approx 50$ Hz. The focal plane was placed at a height of 100 μm above the bottom of the flow chambers (to match our OTV measurements), and swimming tracks within $\pm\ \sim 30$ μm of the focal plane with minimal visual variation in the vertical axis were collected.

Our results consisted of $N = 51$, 53, and 52 tracks of the *cc125*, *cw15*, and *mstg* strains, respectively, with a mean track duration of 0.9 s or 45 beat cycles. From the videos, centers of the cell body were tracked, as shown in Fig. 2 *a*.

We calculated the mean and the maximum advancing speed for each track. Instantaneous velocity for each track (*inset* Fig. 2 *a*) was used to find the maximum advancing speed U_{max}. U_{max} was the median of the peak velocities, as highlighted by the triangles in the inset of Fig. 2 *a*.

For the mean speed, we discarded the oscillatory part of the track, which corresponds to the back-and-forth component of motion, by applying a low-pass filter (<10 Hz), and calculated the mean speed as the arc length of the resulting trajectory divided by the track duration.

To find the beating frequency f_0, we used two methods involving the fast Fourier transformation (FFT) function in MATLAB (The MathWorks, Natick, MA). The first method has been used in previous studies to resolve the beating frequency of both single cells and populations of cells (40–43). This method uses visual vibration of the cell body in the video as input. Practically, sums of pixel value of each frame were first used to construct a time series, which records the bodily vibrations directly resulting from flagellar beating, and f_0 was resolved from the FFT of the time series. To highlight the informative part, we applied a tracking mask that blacked out all but a circular region of radius of ~ 8 μm around the cell center and kept the

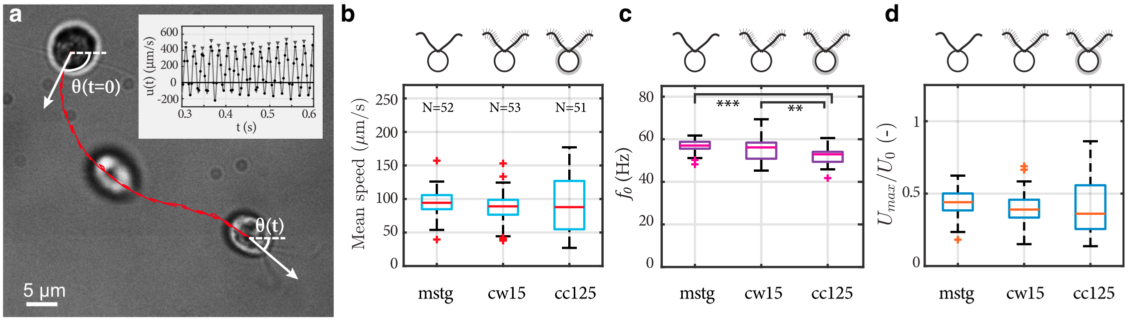

FIGURE 2 Kinematics of freely swimming cells. (*a*) Overlaid image sequence from a typical observation. The time interval between the overlaid snapshots is 200 ms. The red line represents the position of the center of the tracked cell. The white arrows depict the heading angle θ of the cell. The inset shows the instantaneous velocity for the cell in the image with the maximum velocities highlighted by the triangles. (*b–d*) Comparison of (*b*) mean swimming speed, (*c*) beating frequency f_0, and (*d*) maximum swimming speed U_{max} for *mstg*, *cw15*, and *cc125* cells from left to right, respectively. (*d*) U_{max} represents the median of the peak velocities depicted in the inset of (*a*) and is normalized by $U_0 = Lf_0$, where L is flagella length. In (*c*), ** represents $p < 0.01$ and *** represents $p < 0.001$. The distributions in (*b–d*) are compared using the Kruskal-Wallis one-way ANOVA statistical test. For (*b–d*), and all distributions in the subsequent figures, the red lines represent the medians and the bottom and top of the boxes the 25th and 75th interquartiles, respectively. The whiskers represent data between $q_1 - 1.5(q_3 - q_1)$ to $q_3 + 1.5(q_3 - q_1)$, where q_1 and q_3 are the 25th and 75th interquartiles, respectively. Any points outside of this range are marked as red crosses and considered outliers. To see this figure in color, go online.

spectrum higher than 10 Hz for frequency analysis. The second method used FFT of the tracks of the cell centers (*red curve* in Fig. 2 *a*). In the frequency domain, the peaks represented the rate at which the cell body proceeded back and forth along the track. For each track, the sharpest peak between the two methods was reported as f_0 (Fig. 2 *c*). The two methods made use of different input information, namely, the flagellar-beating-induced fluctuation in the cell's shading and the flagellar-beating-induced locomotion. When an observed cell is slightly out of focus or the net forward motion is not substantial, then the FFT of the image results in a sharper peak of f_0. On the other hand, when the cell is in focus and has substantial forward motion, then the FFT of the track results in a sharper peak. However, results of the two methods corroborate each other well.

The turning rate was calculated using the cells' heading direction θ, depicted by the white arrows in Fig. 2 *a*. θ was found from the smoothed trajectory used for the mean speed calculations (using the <10-Hz low-pass filter). The smoothed trajectory did not contain the cells' back-and-forth motion. The turning rate was then calculated as the average of the absolute value of the rate of change of the cells' heading direction or $\overline{|d\theta/dt|}$.

Optical tweezers-based velocimetry

To measure the flow fields around beating cells, we used an OTV method following Wei et al. (33). This method measures the hydrodynamic force on an optically trapped bead in the vicinity of a beating cell. The optical tweezers used in the study are similar to those in Lang et al. (44). Namely, a high-powered laser (1064-nm wavelength) was focused through a water immersion objective (CFI Plan Apo VC 60× NA = 1.20; Nikon) to generate a trapping force $\mathbf{F} = -k\Delta\mathbf{x}$, where $\Delta\mathbf{x}$ is the bead displacement from the center of the trapping laser and k is the trap stiffness. The bead displacement $\Delta\mathbf{x}$ was measured at a sampling frequency of 10 kHz using back focal plane interferometry. The beads had radii of either $a = 1$ or 2.5 μm, with trap stiffness values of $k = 12$–50 pN μm^{-1}.

The flow velocity $\mathbf{u}(t) = (u, v)$ at the location of the trapped bead (Fig. 4 *a*) was determined from the measured bead displacement $\Delta\mathbf{x}$ following the Boussinesq-Basset-Oseen equation. Because the particle Reynolds number$Re_a = \frac{\rho a|\mathbf{u}|}{\mu} \approx 10^{-5} - 10^{-4}$ is low and inertia, added mass, and Basset forces are negligible (45), the Boussinesq-Basset-Oseen equation can be reduced to a first-order equation in which the trapping force $\mathbf{F}$ and hydrodynamic drag are balanced, or $\dot{\Delta\mathbf{x}} + \frac{k}{\zeta}\Delta\mathbf{x} = \mathbf{u}(t)$, where $\zeta = 6\pi\mu a$. Similar methods have been used in previous studies to measure flows in microfluidic devices (46–49).

Numerical simulations

To model the flow fields around individual beating cells, we solved the three-dimensional (3D) Stokes equations using a hybrid boundary element method (BEM) and slender-body approach, similar to Wei et al. (33) and Kim and Karrila (45). The Stokes equations are $\nabla\cdot\mathbf{u} = 0$ and $-\nabla p + \mu\nabla^2\mathbf{u} = 0$, where p is the pressure field. These equations have been found to accurately depict the flow dynamics close to the cell ($y < 0.2\delta$, where $\delta = \sqrt{\nu/f_0}$ and ν is the kinematic viscosity of water) (33), and so, we conducted our simulations within this range.

To capture the fluid-structure interactions for the cell body and glass micropipette, we represented them with a completed double-layer boundary-integral equation (50,51). The singularities of the completion flow were distributed along the centerline of the micropipette. The flagella were represented using slender-body theory with 26 discrete points along each of the flagella's centerline (52). The time dependent motion of each of the 26 discrete points on a beating flagellum were tracked from high-speed videos (~700 Hz), as discussed in High-speed imaging and light microscopy. We then obtained the simulated flow field $\mathbf{u_s}(t) = (u_s, v_s)$, which we compared with the OTV measurements $\mathbf{u}(t) = (u, v)$.

RESULTS AND DISCUSSION

Transmission electron microscopy of flagella and mastigonemes

The *mstg* mutant used in this study carries a mutation in the MST1 gene coding for a mastigoneme-like flagellar protein. TEM was used to confirm the presence of mastigonemes in the *cc125* and *cw15* cells, and their lack in *mstg* cells. Fig. 1, *b–d* show representative TEM images of flagella from *cc125*, *cw15*, and *mstg* cells, respectively. From these images we measured the flagella length $L = 12.0 \pm 1.7$ μm (average ± standard deviation, same for the rest of the text) and flagella radius $r = 0.23 \pm 0.02$ μm. The flagella from *cc125* and *cw15* exhibit two rows of mastigonemes, and the *mstg* flagella appear smooth without any mastigonemes. The mastigonemes on the *cc125* and *cw15* cells appear to be $l_m = 850 \pm 97$ nm in length and $d_m = 16 \pm 3$

nm in diameter, in agreement with previous observations (18), and with densities of 10.1 ± 0.8 mastigonemes per micrometer. These measured values may not accurately represent the physical dimensions of the mastigonemes because the delicate structures may be affected by the staining procedure.

We observed mastigonenemes in 27 out of 28 *cc125* cells and 20 out of 21 *cw15* cells. In contrast, we found mastigonemes for none of the 22 *mstg* cells observed. Moreover, after ~10 times of slant propagation over 18 months, during which the experiments were conducted, we repeated the comparison to confirm whether the genetic mutation was stable. Consistently, all *cc125* ($N = 6$) and *cw15* cells ($N = 6$) showed mastigonemes, whereas none of the *mstg* cells ($N = 6$) did. Therefore, we conclude that the mutation of the MST1 gene was effective and stable.

Using the TEM measurements, we can estimate the possible contribution of these mastigonemes to swimming performance. Following Brennen (20) and Namdeo et al. (22), one can estimate the total drag force on a flagellum, to leading order, as the linear summation of two parts: the drag of the flagellum without mastigonemes and the drag of each mastigoneme. Because the expected drag coefficients depend linearly on length and weakly on radius, hydrodynamic effects of the radius are neglected and only those of the total lengths are compared. The mastigonemes are relatively long ($l_m \sim 0.8$ μm) and are present at high densities (~10 per μm). Their total combined length is an order of magnitude longer than the flagellum (130 μm compared to 12 μm). Therefore, if they would not deflect, we expect free-swimming speed to be significantly affected by their presence (20–22).

Observations of freely swimming cells

After confirmation of the lack of mastigonemes in *mstg* and the presence of mastigonemes in *cc125* and *cw15*, we compared the free-swimming speed, beating frequencies, and turning rates of the three strains. The algae were observed in flow chambers 20 mm wide and 2 mm in height. The observed cells swam 100 μm above the closest wall to minimize any near-field wall effects.

Our observations were made using high-speed videography with spatial resolution of 0.1 μm px^{-1}. The videos were taken at a frame rate of 301 Hz, well above the minimum sampling rate required to resolve their flagella beating at frequencies $f_0 \approx 50$ Hz. An image sequence from a typical observation is shown in Fig. 2 *a*. The average duration of each acquisition was 0.9 s.

The red curve in Fig. 2 *a* depicts the trajectory of the cell body's centroid, and the inset shows a typical plot of the instantaneous swimming speed as a function of time. From the trajectory and instantaneous velocity, we extract the mean swimming speed, beating frequency f_0, and maximum swimming speed U_{max}. The white arrows in Fig. 2 *a* represent the heading direction θ of the swimming cell within the imaging plane, which is used to compute the turning rate.

The swimming trajectories of the cells were helical, as reported before (53,54). Our measurements were two-dimensional projections of this 3D motion, and this is the typical methodology for velocity measurements adopted in the literature (55). This approach and its reliability are detailed in the Supporting Materials and Methods.

Fig. 2 *b* shows the average swimming speed distributions for *mstg*, *cw15*, and *cc125* cells, from left to right, respectively. The distributions in mean speeds are not statistically different between the different cell strains (Kruskal-Wallis one-way analysis of variance (ANOVA), $p > 0.05$). Our observations differ from those reported for cells with mastigonemes removed by an antibody in which a decrease of 20–30% was observed after their removal (35). The mean swimming speed of the wild-type cells measured by Nakamura et al. was 137.3 $\pm$ 16.0 μm s^{-1} (35), whereas ours was 90.2 $\pm$ 39.0 μm s^{-1} (average $\pm$ standard deviation). Although our observed swimming speeds are a bit lower, both observations agree with those previously reported for cells swimming in 3D (110 ± 12 μm s^{-1} (39), 136 ± 12 μm s^{-1} (56), and ~130 μm s^{-1} (57,58)).

The beating frequency f_0 distributions are represented in Fig. 2 *c*. We find that the cell-wall-deficient strains (both *mstg* and *cw15*) exhibit significantly higher beating frequencies ($f_0 = 57.0 \pm 2.9$ and 55.2 ± 5.0 Hz, respectively) when compared to *cc125* cells ($f_0 = 52.2 \pm 4.1$ Hz). The higher frequencies may be a result of the lack of cell wall. The two flagella protrude from the cell wall, and possibly make contact with it while beating. It is possible that either the mechanical confinement or coupling provided by the cell wall influences beating frequency because mechanical coupling between flagella is known to affect their synchronized beating frequency (39,59).

In Fig. 2 *d*, we present the distributions of the maximum speed U_{max}, scaled with the characteristic velocity $U_0 = Lf_0$ to account for the variability in beating frequency and flagella length. The maximal speed U_{max} was computed, for each track, as the median value of the peak velocities of each beat cycle (see *inset* of Fig. 2 *a*). We did not observe any significant differences in U_{max}/U_0 between the strains (Kruskal-Wallis one-way ANOVA, $p > 0.05$). Therefore, even though the *mstg* and *cw15* strains beat faster than *cc125*, they were still propelling themselves at the same maximum speed during their power stroke relative to their flagella beating speed U_0. Previous observations of U_{max} ~320 μm s^{-1} (58) for *cc125* are within the range of our observations of $U_{max} = 254.3 \pm 110.1$ μm s^{-1}.

Incidentally, Fig. S1 shows the turning rate distributions for *mstg*, *cw15*, and *cc125* cells and an image sequence of a representative turning event with a large turning rate. The turning rates were determined from the time derivative

of the heading direction θ or $(d\theta/dt)$. This derivative was computed for every time step, the absolute value of the derivative is taken, and then an average value for the duration of each acquisition was reported as the turning rate or $\overline{|d\theta/dt|}$. We found that *mstg* had a significantly higher turning rate compared to *cw15* and *cc125*. However, upon close inspection of the recordings, we see the increased turning to be a direct result of a decrease in symmetry between the beating of the two flagella, either because of slipping (Video S1), in which the flagella briefly lose their synchrony, or because of an asymmetry in the flagellar kinematics (Video S2), in which one flagellum swipes a larger area than the other.

From our observations of freely swimming cells, we find that mastigonemes do not enhance swimming performance. We find no significant differences in either mean or maximum forward-swimming speeds between cells with or without mastigonemes. Our results differ from the observations of Nakamura et al. (35), which used a monoclonal antibody to remove the mastigonemes and observed a decrease of the swimming velocity for the cells without mastigonemes.

Comparison of flagellar waveform

The swimming velocity of flagellated organisms is determined by both the hydrodynamic drag on the flagella and the kinematics of the flagellar deformation. Both need to be fully characterized to assess the hydrodynamic effect of mastigonemes.

Therefore, we proceed by analyzing the flagellar gait kinematics to determine whether strain mutations and their subsequent morphological differences affect beating patterns. High-speed videos of flagellar beating of cells held by a micropipette were recorded at 700 Hz. Typical flagellar waveforms are shown with respect to the cell's frame of reference (x, y) in Fig. 3, *a–c*. The cell's frame of reference is defined schematically in the inset of Fig. 1 *a*. To quantify gait kinematics, we parameterized the flagellar waveform of cells from each strain following the method used in Geyer et al. (60) and similar to VanderWaal et al. (61) and Bottier et al. (62).

This parameterization divides the motion of a flagellum into a static and dynamic component, where the flagellar motion is represented as a circular arc (static) and a sinusoidal traveling wave (dynamic). For the analysis, we measured the local angle ψ tangent to the flagellum for 26 discrete points along the flagellar arc length s for each recorded frame at time t, which resulted in a relationship for $\psi(s,t)$. The parameters s and ψ are defined in Fig. 1 *a*. Typical flagellar waveforms are shown with respect to the local frame of reference (s, ψ) in Fig. 3, *d–f*. The static component of the motion is $\psi_1(s) = Cs$, where C represents the mean curvature of the flagellum. The dynamic component of the motion is $\psi_2(s,t) = A\sin(2\pi(f_0 t + s/\lambda))$, where A is the amplitude of the traveling wave, f_0 is the beating frequency, and λ is the wavelength.

Combining the static $\psi_1(s)$ and dynamic $\psi_2(s,t)$ components yields an expression for the tangent angle along the flagellar length, or $\psi(s, t) = Cs + A\sin(2\pi(f_0 t + s/\lambda))$. The parameters C, A, and λ were extracted for each

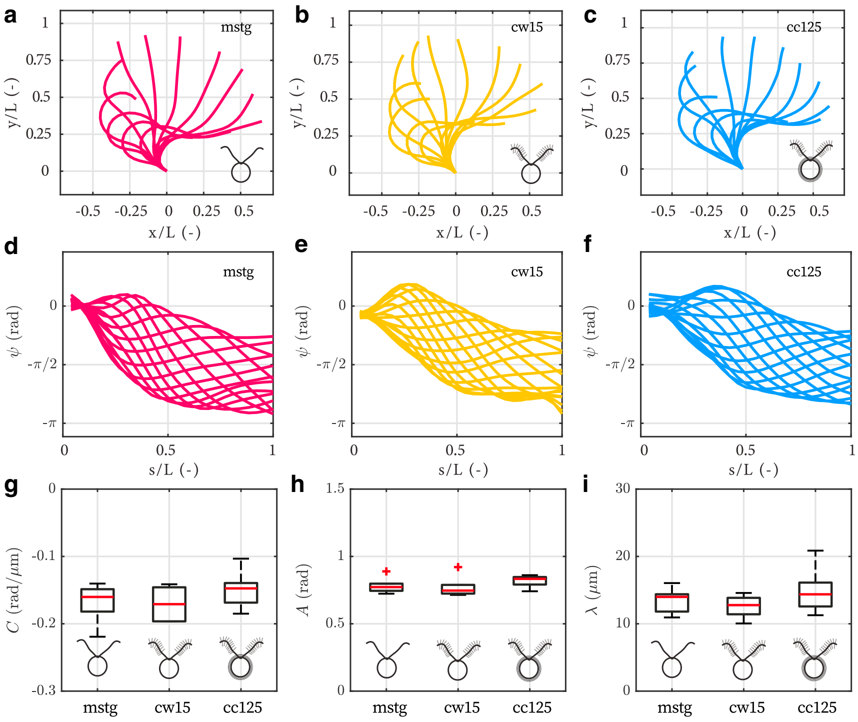

FIGURE 3 Flagellar gait kinematics. (*a–c*) Representative flagellar waveforms with respect to the cell's frame of reference (*x*, *y*), defined in Fig. 1 *a*, for (*a*) *mstg*, (*b*) *cw15*, and (*c*) *cc125* cells. (*d–f*) Representative flagellar waveforms with respect to the local frame of reference (s, ψ), defined in Fig. 1 *a*, for (*d*) *mstg*, (*e*) *cw15*, and (*f*) *cc125* cells. Here, ψ is the local tangent angle, and s is the arc length along the centerline of the flagellum. The flagellum length L is used to normalize spatial coordinates x, y, and s. (*g–i*) Comparison of fitting parameters for flagellar waveforms of *mstg*, *cw15*, and *cc125* cells from left to right, respectively. The parameters are (*g*) average curvature C, (*h*) traveling wave amplitude A, and (*i*) wavelength λ. The sample size for (*g–i*) is $N = 6$ for each strain. To see this figure in color, go online.

flagellum of $N = 6$ cells per strain. For all three strains, we compared the mean curvature C (Fig. 3 *g*), traveling wave amplitude A (Fig. 3 *h*), and wavelength λ (Fig. 3 *i*). In each panel, *mstg*, *cw15*, and *cc125* are displayed respectively from left to right. For all three waveform parameters, we find no statistical differences between the strains (Kruskal-Wallis one-way ANOVA, $p > 0.05$).

The mean curvature is $C = -0.17 \pm 0.03$, -0.17 ± 0.02, and -0.15 ± 0.02 rad μm^{-1} (average ± standard deviation) for *mstg*, *cw15*, and *cc125* cells, respectively. The values of C for *cc125* cells are in agreement with those of previous studies (-0.17 ± 0.005 rad μm^{-1} (60,63) and -0.21 ± 0.07 rad μm^{-1} (62)). The traveling wave amplitude is $A = 0.78 \pm 0.05$, 0.77 ± 0.07, and 0.82 ± 0.04 rad (average ± standard deviation) for *mstg*, *cw15*, and *cc125* cells, respectively. The values of A for *cc125* are also in agreement with those of previous studies (1.08 ± 0.09 rad (60,63) and ~0.89 ± 0.06 rad (62)). Finally, the wavelength is $\lambda = 13.5 \pm 1.7$, 12.6 ± 1.5, and 14.9 ± 3.1 μm (average ± standard deviation) for *mstg*, *cw15*, and *cc125* cells, respectively. Values of λ for *cc125* cells are again in agreement with previous studies (15.1 ± 2.0 μm (60,63)).

In conclusion, because there are no significant differences for all three parameters that characterize the flagellar waveform, the flagellar gait kinematics and deformations appear unaffected by the presence of mastigonemes.

Optical tweezers-based velocimetry

After having observed no significant differences in the free-swimming characteristics or flagellar kinematics of cells with and without mastigonemes, we directly probed the hydrodynamics of captured cells using OTV (33). This technique allowed us to locally measure the flow field generated by beating flagella with and without mastigonemes.

A schematic depicting our OTV experiments is shown in Fig. 4 *a*, with a picture from our experiments in the inset at the top right. A polystyrene bead of diameter 2 or 5 μm was trapped in the vicinity of a flagellum. We measured the local two-dimensional flow field $\mathbf{u}(t) = (u, v)$, u (axial) and v (lateral), generated by the cell at the location of the bead. The flow field was recorded at increasing lateral distances from the cell by moving the bead along the y-axis. This procedure was previously implemented to study the flow around *cc125* cells (33). We also defined the flagellar phase ϕ to represent the flagellar shapes during a beat cycle. The most forward-reaching shape, as shown in the inset of Fig. 4 *a*, was set as the beginning of a cycle ($\phi = 0$). Typically, the power stroke ends at $\phi \approx \pi$, shown in Fig. 4 *a*.

The periodic signal of axial u and lateral v velocities were recorded over successive flagellar beat cycles with a high temporal resolution of 10 kHz; see Fig. 4, *b* and *c*. To compare the hydrodynamics of individual cells across strains, we reconstructed an average beat cycle for each cell and extracted the amplitude of the oscillations and the time-averaged flow velocity generated in the axial ($\Delta u'$ and $\bar{u}$, respectively) and lateral directions ($\Delta v'$ and $\bar{v}$, respectively) for each individual cell.

Fig. 4, *d* and *e* represent the flow velocity of a typical beat cycle, constructed as the median over 50 cycles. The asymmetry in the power-recovery stroke is clearly visible in the axial flow u, with the power stroke generating a strong positive flow for the first half of the cycle ($\phi < \pi$), and the recovery stroke subsequently generating a weaker negative flow. The flow amplitudes ($\Delta u'$ and $\Delta v'$) and average flows

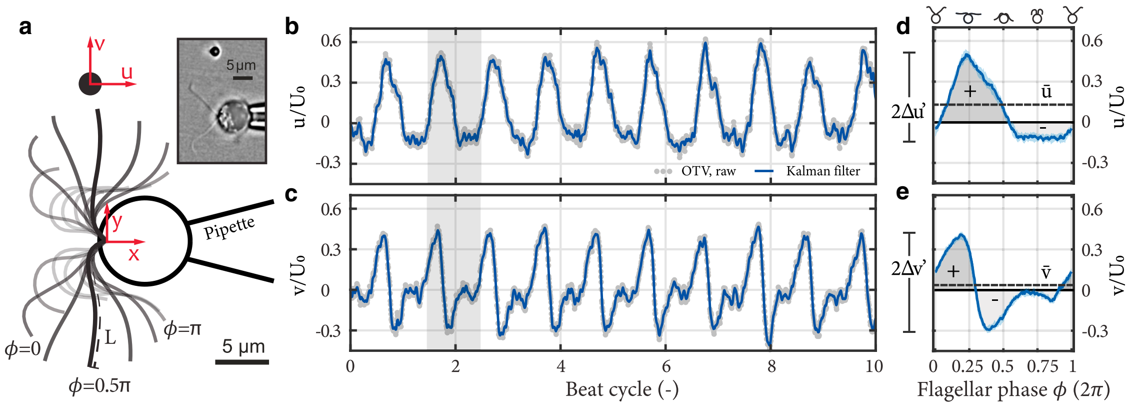

FIGURE 4 Optical tweezers-based velocimetry (OTV). (*a*) Schematic of the OTV measurements with an algal cell captured by a micropipette and optically trapped bead to measure flow velocities u and v. Inset shows a light microscope image from a typical experiment. Scale bars, 5 μm. (*b* and *c*) Periodic signals of (*b*) axial u and (*c*) lateral v flow velocities as a function of beat cycle. The gray points are raw OTV data, and the blue solid curve is data filtered with a Kalman filter. A typical beat cycle is shaded; it begins with the most forward-reaching flagellar shapes, defined as flagellar phase $\phi = 0$, shown in the inset of (*a*).The median cycle is constructed based on ~50 cycles and shown in (*d* and *e*). (*d* and *e*) Flow velocities (*d*) u and (*e*) v over one average beat cycle. In (*d*), the dark gray "+" represents the power stroke, when propulsive flows are generated. The light gray "−" represents the recovery stroke when the flagella move opposite to the swimming direction. In (*e*), the + and −, together with their shadings, mark, respectively, the propulsion outwards and inwards in the cell's lateral direction. The flow amplitudes $\Delta u'$ and $\Delta v'$ are depicted on the left. The dashed lines represent the average velocities $\bar{u}$ and $\bar{v}$. The data shown in (*b*)–(*e*) are from a *cc125* cell. To see this figure in color, go online.

($\overline{u}$ and $\overline{v}$) are extracted from the typical beat cycle as represented on Fig. 4, *d* and *e*. Schematics representing the shapes at each flagellar phase ϕ are displayed in the upper horizontal axis of Fig. 4 *d*.

We use the flow parameters $\overline{u}$, $\overline{v}$, $\Delta u'$, and $\Delta v'$ to investigate the flow field around beating cells and compare between the three strains to determine the hydrodynamic effects of mastigonemes. The distributions of average flows and amplitude of flow oscillations are reported in Fig. 5. The distance y from the cell is scaled by the diffusive length scale $\delta = \sqrt{\nu/f_0}$, where ν is the kinematic viscosity of water. The insets present the same data in log-log scale and highlight the rate of spatial decay of the velocity field. The beating flagella generate a flow, which is significantly stronger in the axial x-direction Fig. 5 *a* compared with the lateral y-direction Fig. 5 *b*. The magnitude of the average flow decays in $\sim 1/y$, as expected from the stokeslet flow (Fig. 5, *a* and *b*). The amplitude of the flow oscillations $\Delta u'$ and $\Delta v'$ is large in the vicinity of the flagella but decays in $\sim 1/y^3$, faster than the average flow (33); see Fig. 5, *c* and *d*.

The spatial distributions of average velocity and amplitude of oscillation reveal no significant differences in the flow fields generated by the three different strains; see Fig. 5. The measurements were performed for each strain for different cells with $N = 9 - 14$. The variability of the measurements for a given strain is represented by the interquartile range in Fig. 5 and is comparable to the variability observed in swimming velocities; see Fig. 2. The differences in the flow fields generated by *mstg*, *cw15*, and *cc125* are within the variability observed within a given strain. Overall, experimental measurements do not evidence an increase in flow generation because of the presence of mastigonemes.

Comparisons with hydrodynamic modeling

To directly quantify the hydrodynamic effect of mastigonemes, we performed computational fluid dynamic simulations to compute the flow field close to the flagella and directly compared them with our time-resolved flow measurements. To account for individual cell to cell variations between our experiments, we first tracked the flagellar shapes for each recording (see Fig. 3) and used the deformations as boundary conditions for the simulation. The flagellar kinematics are tracked using high-speed videography ($\sim$700 Hz). The flow is simulated by solving Stokes equations, which have been shown previously to accurately model fluid flows in the near-field, for $y < 0.2\delta$ (33). Our hydrodynamic model assumes the flagella to be cylindrical, and so, without mastigonemes; therefore, agreement with measurements from *mstg* and disagreement with measurements from *cw15* and *cc125* would reveal the hydrodynamic contributions from mastigonemes.

Fig. 6, *a–c* show the relationship between flow velocity, u (left) and v (right), and beat cycle for (Fig. 6 *a*) *mstg*, (Fig. 6

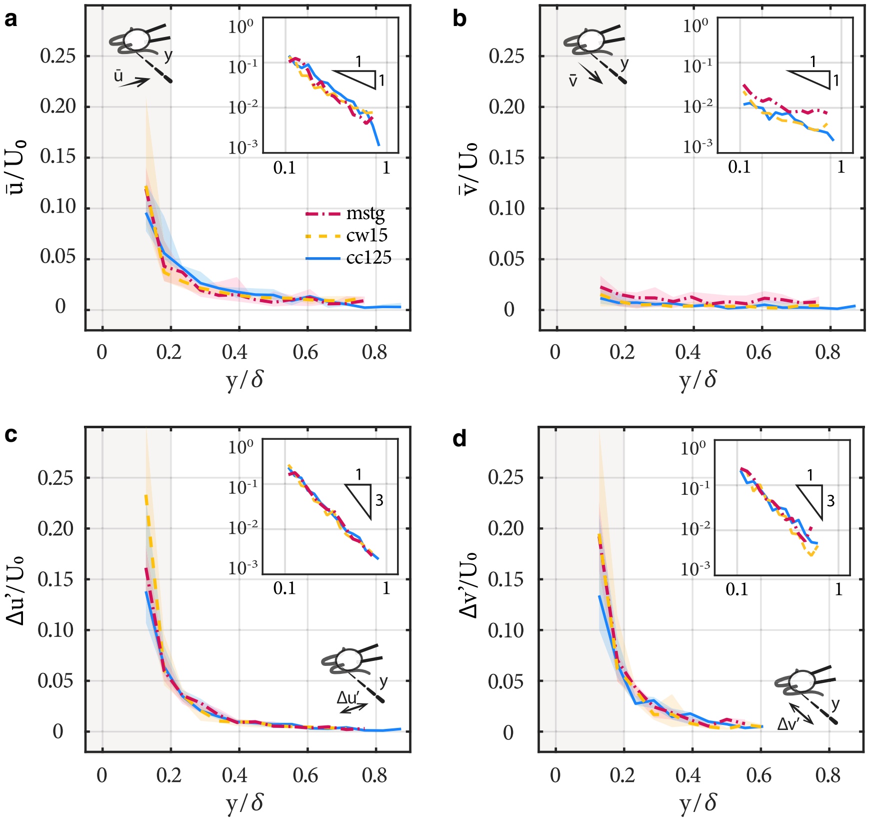

FIGURE 5 Hydrodynamics of beating flagella from optical tweezers-based velocimetry (OTV). (*a* and *b*) Relationship between average (*a*) axial $\overline{u}$ and (*b*) lateral $\overline{v}$ flows and distance y from the midplane of the cell. (*c* and *d*) Relationship between flow amplitudes in (*c*) axial $\Delta u'$ and (*d*) lateral $\Delta v'$ directions and distance y. The red dash-dot line is for *mstg* ($N = 9$), the yellow dashed line is for *cw15* ($N = 11$), and the solid blue line is for *cc125* ($N = 14$) cells. The lines represent the median, whereas the shaded region represents the interquartile. The distance y is normalized by $\delta = \sqrt{\nu/f_0}$, where ν is the kinematic viscosity of water. Insets show data plotted in log-log scale to highlight the rates of spatial decay for the flows. To see this figure in color, go online.

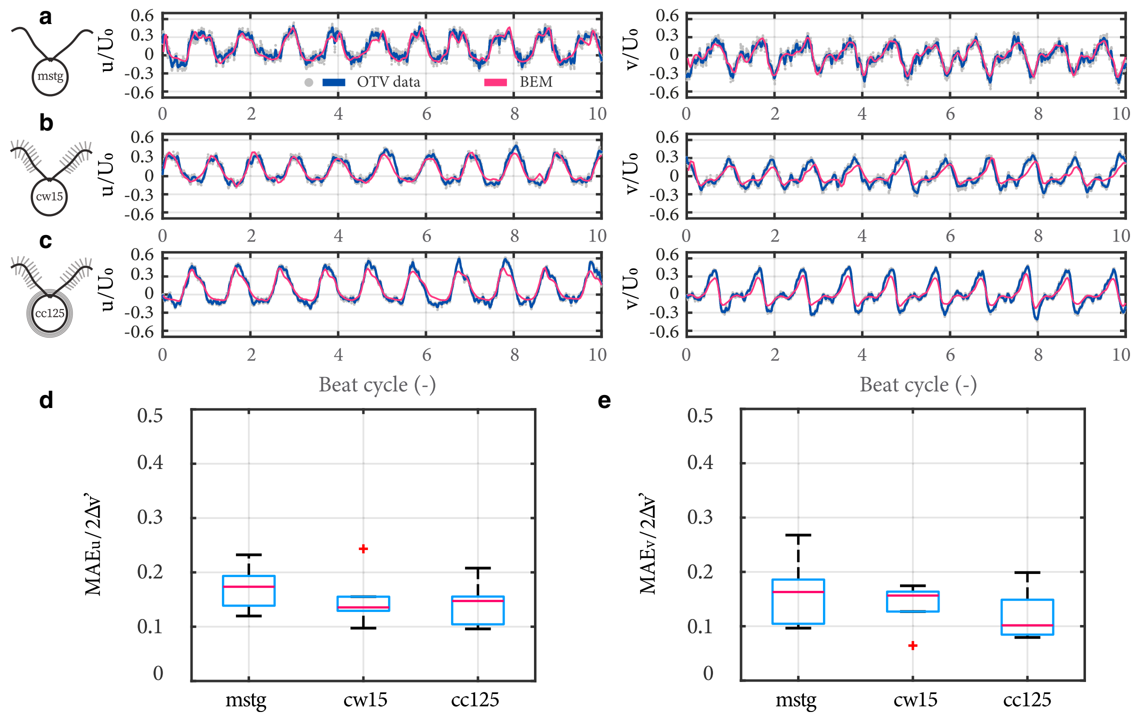

FIGURE 6 Near-field hydrodynamics of beating flagella. (*a–c*) Axial u (*left*) and lateral v (*right*) flow as a function of beat cycle for a typical (*a*) *mstg*, (*b*) *cw15*, and (*c*) *cc125* cell. The red solid curve is the prediction from hydrodynamic modeling (BEM). (*d* and *e*) Comparison of MAE of BEM predictions for (*d*) axial flows MAE_u and (*e*) lateral flows MAE_v, for *mstg*, *cw15*, and *cc125* strains from left to right, respectively. The data represented are from within ~20 μm of a beating cell ($y < 0.2\delta$), or the lightly shaded areas in Fig. 5. The sample size for (*d*) and (*e*) is $N = 6$ for each strain. To see this figure in color, go online.

b) *cw15*, and (Fig. 6 *c*) *cc125* cells. The red curves represent our simulations using a hybrid BEM and slender-body approach to solve Stokes equations around beating cells (33,45,51,52). Video S3 shows a representative flagellar stroke from experiments, followed by the flow field predicted from the simulations.

Because our simulations assume that the flagella do not have mastigonemes, we begin by comparing with experiments for *mstg*. From Fig. 6 *a*, we observe good agreement between measurements and BEM. Quantitatively, we compute the mean absolute error (MAE), which is the average deviation of the instantaneous flow velocity between BEM and experiments, or$MAE_{\mathbf{u}} = \sum_{i=1}^{n} \left| \mathbf{u}_{\mathbf{s,i}} - \mathbf{u_i} \right| / n$, where $\mathbf{u}_{\mathbf{s,i}}$ is the instantaneous velocity predicted by simulation and n is the total number of instantaneous points measured/simulated. In Fig. 6, *d* and *e*, we show the error of our simulations in the axial and lateral direction for *mstg* normalized by the flow amplitude, or (Fig. 6 *d*) $MAE_u/2\Delta u'$ and (Fig. 6 *e*) $MAE_v/2\Delta v'$, respectively. The error values are around 0.15. Therefore, our simulations accurately capture the flow field generated by beating flagella.

After validation of our simulations with the mastigonemeless mutant *mstg*, we proceed by comparing simulations and experiments for *cw15* and *cc125* cells. Similar to *mstg*, we find good agreement between experiments and BEM in Fig. 6, *b* and *c*, which are confirmed quantitatively in Fig. 6, *d* and *e*. Moreover, we find no statistical differences between errors in simulation predictions across the three strains (Kruskal-Wallis one-way ANOVA, $p > 0.05$).

If the mastigonemes contributed to the hydrodynamics, we would have expected better agreement between measurements and simulations for the *mstg* cells because our modeled flagella are smooth. Instead, we find no differences between strains, and, in all cases, the observed hydrodynamics around beating cells are well captured by representing the flagella as smooth, i.e., neglecting the mastigonemes.

This is unlike what we expect if mastigonemes are rigid and dense and increase the effective width of a flagellum. By direct computation using BEM, we solve Stokes equations around slender flagella of different cross-sectional geometries and obtain their drag coefficients, see Supporting Materials and Methods for details. Assuming the mastigonemes do not deform, we estimate the drag coefficient of a straight flagellum of length $L = 12$ μm, width $w = 2(r + l_m) = 2$ μm, and thickness $h = 2r$. The width w accounts for the two rows of mastigonemes of length l_m observed in Fig. 1 *b*. The geometry is shown in Fig. S2. We compare this value with the drag coefficient of a straight and smooth flagellum of length $L = 12$ μm and diameter $2r = 0.5$ μm.

We find the drag increase is 50% for flow in the direction perpendicular to the mastigonemes, and 30% for flow in the direction parallel to them. The 50% increase is comparable with estimates of the drag coefficient around a cylinder of increased diameter from $2r = 0.5\ \mu m$ to $2(r + l_m) = 2\ \mu m$ (64). Alternatively, for an undulating swimmer without a separate cell body (*C. elegans*), a 30% reduction in swimming speed is predicted for the same increase in diameter (24). Therefore, according to both models, the hydrodynamic effect of straight and rigid mastigonemes would be significant and clearly measurable given the accuracy of the OTV technique.

Overall, from our OTV observations and hydrodynamics modeling, we conclude that the mastigonemes do not contribute to the flows generated by beating flagella and do not have a direct hydrodynamic effect. There are no differences between the axial u or lateral flows v generated per stroke by *mstg*, *cw15*, or *cc125* cells. Additionally, our hydrodynamic model, which assumes smooth flagella without mastigonemes, accurately represents the fluid dynamics around both cells with mastigonemes (*cc125* and *cw15*) and cells without (*mstg*).

Since we do not observe any hydrodynamic differences because of the presence of mastigonemes, we can conclude that the increased turning rate in *mstg* is not due to a direct hydrodynamic effect of the mastigonemes and, more likely, results from either differences in flagella swiping areas or slipping events (see Observations of freely swimming cells). These behavioral differences would require further studies to determine whether and how they are linked to the presence of mastigonemes.

Our results refute the hypothesis currently accepted in the literature that the fibrous mastigonemes increase the effective area of *C. reinhardtii*'s flagella during their beating strokes (18). A possible explanation may be the flexibility of the mastigonemes of *C. reinhardtii*. Because they are thin and of the fibrous type (18), they may bend and deflect when the flagella moves through the fluid. As pointed out by Brennen (20) and Namdeo et al. (22), if the flexibility of the mastigonemes is below a critical value, then they would not contribute to the drag of the flagellum, and the drag force would be equivalent to that of a smooth flagellum.

CONCLUSIONS

Our study thoroughly investigated the possible hydrodynamic contribution of the mastigonemes of *C. reinhardtii* using a mutant lacking these structures. In conclusion, we confirmed through TEM imaging that the *mstg* mutant used in our study lacks mastigonemes, whereas the *cw15* and *cc125* cells possess them. From observations of freely swimming cells, we find that mastigonemes do not increase swimming velocity. Furthermore, mastigonemes do not affect flagellar gait kinematics. Finally, from our OTV measurements, we find that mastigonemes do not help produce larger hydrodynamic forces or propulsive flows per stroke, as has been previously hypothesized (18,34,35). Future studies into the bending stiffness of *C. reinhardtii* mastigonemes may help confirm whether mastigonemes bend and deflect during swimming, which render them useless for hydrodynamic enhancement. Additionally, future studies into the effects of mastigonemes on swimming behavior could shed light on functions of mastigonemes other than mere hydrodynamics. Overall, our study found that mastigonemes do not contribute to the swimming propulsion of *C. reinhardtii*. Their function still remains enigmatic. More studies into their contributions to sensing, adhesion, or feeding may reveal their function and evolution.

SUPPORTING MATERIAL

Supporting Material can be found online at https://doi.org/10.1016/j.bpj.2020.05.003.

AUTHOR CONTRIBUTIONS

D.W. and G.J.A. performed research. All authors designed research, analyzed data, and wrote the manuscript. D.T. and M.-E.A.-T. supervised the work and are both corresponding authors.

ACKNOWLEDGMENTS

The authors thank B. Beaumont for a useful suggestion and R. Kieffer for technical support.

The work was supported by the European Research Council (Starting Grant no. 716712), the Netherlands Organisation for Scientific Research (Frontiers of Nanoscience program), and the European Union's Horizon 2020 Research and Innovation Programme under the Marie Skłodowska-Curie Grant Agreement no. 707404.

REFERENCES

1. Bejan, A. 1990. Theory of heat transfer from a surface covered with hair. *J. Heat Transfer.* 112:662–667.
2. Casas, J., and O. Dangles. 2010. Physical ecology of fluid flow sensing in arthropods. *Annu. Rev. Entomol.* 55:505–520.
3. Amador, G. J., and D. L. Hu. 2015. Cleanliness is next to godliness: mechanisms for staying clean. *J. Exp. Biol.* 218:3164–3174.
4. Autumn, K., M. Sitti, ..., R. J. Full. 2002. Evidence for van der Waals adhesion in gecko setae. *Proc. Natl. Acad. Sci. USA.* 99:12252–12256.
5. Swaddle, J. P., M. S. Witter, ..., P. McCowen. 1996. Plumage condition affects flight performance in common starlings: implications for developmental homeostasis, abrasion and moult. *J. Avian Biol.* 27:103–111.
6. Sato, K., H. Takahashi, ..., I. Shimoyama. 2013. Effectiveness of bristled wing of thrips. In 2013 IEEE 26th International Conference on Micro Electro Mechanical Systems (MEMS). IEEE, pp. 21–24.
7. Rubenstein, D. I., and M. Koehl. 1977. The mechanisms of filter feeding: some theoretical considerations. *Am. Nat.* 111:981–994.
8. Nasto, A., P.-T. Brun, and A. E. Hosoi. 2018. Viscous entrainment on hairy surfaces. *Phys. Rev. Fluids.* 3:024002.
9. Vogel, S. 1983. How much air passes through a silkmoth's antenna? *J. Insect Physiol.* 29:597–602.

10. Amador, G. J., W. Mao, …, D. L. Hu. 2015. Eyelashes divert airflow to protect the eye. *J. R. Soc. Interface.* 12:20141294.

11. Amador, G. J., F. Durand, …, D. L. Hu. 2015. Insects have hairy eyes that reduce particle deposition. *Eur. Phys. J. Spec. Top.* 224:3361–3377.

12. Spedding, G. 1987. The wake of a kestrel (*Falco tinnunculus*) in flapping flight. *J. Exp. Biol.* 127:59–78.

13. Pitelka, D. R. 2013. Electron-Microscopic Structure of Protozoa: International Series of Monographs on Pure and Applied Biology, First Edition. Elsevier, Oxford, UK.

14. Jahn, T. L., M. D. Lanoman, and J. R. Fonseca. 1964. The mechanism of locomotion of flagellates. II. Function of the mastigonemes of *Ochromonas*. *J. Protozool.* 11:291–296.

15. Ringo, D. L. 1967. Flagellar motion and fine structure of the flagellar apparatus in *Chlamydomonas*. *J. Cell Biol.* 33:543–571.

16. Loiseaux, S., and J. A. West. 1970. Brown algal mastigonemes: comparative ultrastructure. *Trans. Am. Microsc. Soc.* 89:524–532.

17. Bouck, G. B. 1971. The structure, origin, isolation, and composition of the tubular mastigonemes of the Ochromas flagellum. *J. Cell Biol.* 50:362–384.

18. Bloodgood, R. A. 2009. The Chlamydomonas flagellar membrane and its dynamic properties. The Chlamydomonas Sourcebook, Second Edition. Elsevier, pp. 309–368.

19. Taylor, G. I. 1951. Analysis of the swimming of microscopic organisms. *Proc. R. Soc. Lond. A.* 209:447–461.

20. Brennen, C. 1976. Locomotion of flagellates with mastigonemes. *J. Mechanochem. Cell Motil.* 3:207–217.

21. Holwill, M. E., and M. A. Sleigh. 1967. Propulsion by hispid flagella. *J. Exp. Biol.* 47:267–276.

22. Namdeo, S., S. N. Khaderi, …, P. R. Onck. 2011. Swimming direction reversal of flagella through ciliary motion of mastigonemes. *Biomicrofluidics.* 5:34108–3410815.

23. Tottori, S., and B. J. Nelson. 2013. Artificial helical microswimmers with mastigoneme-inspired appendages. *Biomicrofluidics.* 7:61101.

24. Berman, R., O. Kenneth, …, A. Leshansky. 2013. Undulatory locomotion of finite filaments: lessons from *Caenorhabditis elegans*. *New J. Phys.* 15:075022.

25. Childress, S. 1981. Mechanics of Swimming and Flying. Cambridge University Press, New York, NY.

26. Merchant, S. S., S. E. Prochnik, …, A. R. Grossman. 2007. The *Chlamydomonas* genome reveals the evolution of key animal and plant functions. *Science.* 318:245–250.

27. Polin, M., I. Tuval, …, R. E. Goldstein. 2009. *Chlamydomonas* swims with two "gears" in a eukaryotic version of run-and-tumble locomotion. *Science.* 325:487–490.

28. Drescher, K., R. E. Goldstein, …, I. Tuval. 2010. Direct measurement of the flow field around swimming microorganisms. *Phys. Rev. Lett.* 105:168101.

29. Tam, D., and A. E. Hosoi. 2011. Optimal feeding and swimming gaits of biflagellated organisms. *Proc. Natl. Acad. Sci. USA.* 108:1001–1006.

30. Kurtuldu, H., D. Tam, …, J. P. Gollub. 2013. Flagellar waveform dynamics of freely swimming algal cells. *Phys. Rev. E Stat. Nonlin. Soft Matter Phys.* 88:013015.

31. Brumley, D. R., K. Y. Wan, …, R. E. Goldstein. 2014. Flagellar synchronization through direct hydrodynamic interactions. *eLife.* 3:e02750.

32. Sartori, P., V. F. Geyer, …, J. Howard. 2016. Dynamic curvature regulation accounts for the symmetric and asymmetric beats of *Chlamydomonas* flagella. *eLife.* 5:e13258.

33. Wei, D., P. G. Dehnavi, …, D. Tam. 2019. Is the zero Reynolds number approximation valid for ciliary flows? *Phys. Rev. Lett.* 122:124502.

34. Bouck, G. 1972. Architecture and assembly of mastigonemes. *In* Advances in Cell and Molecular Biology E. J. Dupraw, ed. . Academic Press, pp. 237–271.

35. Nakamura, S., G. Tanaka, …, O. Nikaido. 1996. Assembly and function of *Chlamydomonas* flagellar mastigonemes as probed with a monoclonal antibody. *J. Cell Sci.* 109:57–62.

36. Li, X., W. Patena, …, M. C. Jonikas. 2019. A genome-wide algal mutant library and functional screen identifies genes required for eukaryotic photosynthesis. *Nat. Genet.* 51:627–635.

37. Li, X., R. Zhang, …, M. C. Jonikas. 2016. An indexed, mapped mutant library enables reverse genetics studies of biological processes in *Chlamydomonas reinhardtii*. *Plant Cell.* 28:367–387.

38. Zhang, R., W. Patena, …, M. C. Jonikas. 2014. High-throughput genotyping of green algal mutants reveals random distribution of mutagenic insertion sites and endonucleolytic cleavage of transforming DNA. *Plant Cell.* 26:1398–1409.

39. Quaranta, G., M.-E. Aubin-Tam, and D. Tam. 2015. Hydrodynamics versus intracellular coupling in the synchronization of eukaryotic flagella. *Phys. Rev. Lett.* 115:238101.

40. Sakakibara, H., and R. Kamiya. 1989. Functional recombination of outer dynein arms with outer arm-missing flagellar axonemes of a *Chlamydomonas* mutant. *J. Cell Sci.* 92:77–83.

41. Takada, S., and R. Kamiya. 1997. Beat frequency difference between the two flagella of *Chlamydomonas* depends on the attachment site of outer dynein arms on the outer-doublet microtubules. *Cell Motil. Cytoskeleton.* 36:68–75.

42. Kamiya, R. 2000. Analysis of cell vibration for assessing axonemal motility in *Chlamydomonas*. *Methods.* 22:383–387.

43. Okita, N., N. Isogai, …, K. Yoshimura. 2005. Phototactic activity in *Chlamydomonas* 'non-phototactic' mutants deficient in Ca2+-dependent control of flagellar dominance or in inner-arm dynein. *J. Cell Sci.* 118:529–537.

44. Lang, M. J., C. L. Asbury, …, S. M. Block. 2002. An automated two-dimensional optical force clamp for single molecule studies. *Biophys. J.* 83:491–501.

45. Kim, S., and S. J. Karrila. 2013. Microhydrodynamics: Principles and Selected Applications. Dover Publications, Mineola, NY.

46. Nemet, B. A., and M. Cronin-Golomb. 2002. Microscopic flow measurements with optically trapped microprobes. *Opt. Lett.* 27:1357–1359.

47. Knöner, G., S. Parkin, …, H. Rubinsztein-Dunlop. 2005. Characterization of optically driven fluid stress fields with optical tweezers. *Phys. Rev. E Stat. Nonlin. Soft Matter Phys.* 72:031507.

48. Kabir, A. M. R., D. Inoue, …, A. Kakugo. 2015. Drag force on micron-sized objects with different surface morphologies in a flow with a small Reynolds number. *Polym. J.* 47:564–570.

49. Almendarez-Rangel, P., B. Morales-Cruzado, …, F. G. Pérez-Gutiérrez. 2018. A microflow velocity measurement system based on optical tweezers: a comparison using particle tracking velocimetry. *European Journal of Mechanics B.* 72:561–566.

50. Power, H., and G. Miranda. 1987. Second kind integral equation formulation of Stokesâ€™ flows past a particle of arbitrary shape. *SIAM J. Appl. Math.* 47:689–698.

51. Keaveny, E. E., and M. J. Shelley. 2011. Applying a second-kind boundary integral equation for surface tractions in stokes flow. *J. Comput. Phys.* 230:2141–2159.

52. Keller, J. B., and S. I. Rubinow. 1976. Slender-body theory for slow viscous flow. *J. Fluid Mech.* 75:705–714.

53. Isogai, N., R. Kamiya, and K. Yoshimura. 2000. Dominance between the two flagella during phototactic turning in *Chlamydomonas*. *Zool. Sci.* 17:1261–1266.

54. Crenshaw, H. C. 2015. A new look at locomotion in microorganisms: rotating and translating. *Am. Zool.* 36:608–618.

55. Leptos, K. C., J. S. Guasto, …, R. E. Goldstein. 2009. Dynamics of enhanced tracer diffusion in suspensions of swimming eukaryotic microorganisms. *Phys. Rev. Lett.* 103:198103.

56. Yagi, T., I. Minoura, …, R. Kamiya. 2005. An axonemal dynein particularly important for flagellar movement at high viscosity. Implications

from a new Chlamydomonas mutant deficient in the dynein heavy chain gene DHC9. *J. Biol. Chem.* 280:41412–41420.

57. Wakabayashi, K., T. Ide, and R. Kamiya. 2009. Calcium-dependent flagellar motility activation in *Chlamydomonas reinhardtii* in response to mechanical agitation. *Cell Motil. Cytoskeleton.* 66:736–742.

58. Qin, B., A. Gopinath, …, P. E. Arratia. 2015. Flagellar kinematics and swimming of algal cells in viscoelastic fluids. *Sci. Rep.* 5:9190.

59. Wan, K. Y., and R. E. Goldstein. 2016. Coordinated beating of algal flagella is mediated by basal coupling. *Proc. Natl. Acad. Sci. USA.* 113:E2784–E2793.

60. Geyer, V. F., P. Sartori, …, J. Howard. 2016. Independent control of the static and dynamic components of the *Chlamydomonas* flagellar beat. *Curr. Biol.* 26:1098–1103.

61. VanderWaal, K. E., R. Yamamoto, …, M. E. Porter. 2011. *bop5* Mutations reveal new roles for the IC138 phosphoprotein in the regulation of flagellar motility and asymmetric waveforms. *Mol. Biol. Cell.* 22:2862–2874.

62. Bottier, M., K. A. Thomas, …, P. V. Bayly. 2019. How does cilium length affect beating? *Biophys. J.* 116:1292–1304.

63. Brokaw, C. J., and D. J. Luck. 1983. Bending patterns of *chlamydomonas* flagella I. Wild-type bending patterns. *Cell Motil.* 3:131–150.

64. Cox, R. G. 1970. The motion of long slender bodies in a viscous fluid Part 1. General theory. *J. Fluid Mech.* 44:791–810.

Biophysical Journal, Volume *118*

Supplemental Information

Fibrous Flagellar Hairs of *Chlamydomonas reinhardtii* Do Not Enhance Swimming

Guillermo J. Amador, Da Wei, Daniel Tam, and Marie-Eve Aubin-Tam

Supplementary Information: Fibrous flagellar hairs of *Chlamydomonas reinhardtii* do not enhance swimming

G.J. Amador, D. Wei, D. Tam, M.E. Aubin-Tam

1 Free swimming helical trajectory

For our measurements, we observed the 2D projections of the cells' 3D helical trajectories. As such, we may underestimate the trajectory length.

Here we use the parametric equations for a 3D helix with x the translational direction, to estimate the error in 2D measurements:

$$\begin{aligned} x(t') &= b\cos(t'), \\ y(t') &= ct', \\ z(t') &= b\sin(t'), \end{aligned} \tag{1}$$

Here, t' ranges from 0 to 2π for a single cycle, b is the radius of the helix, and c represents its wavelength. Following previous measurements [1, 2], we assume $b = 5$ μm. The wavelength c is found using the trajectory lengths we measured for the 2D projections assuming a helix frequency of 2 Hz [1, 2]. We calculate the 2D trajectory length L_{2D} as follows:

$$L_{2D} = \int_0^{2\pi} \sqrt{\left(\frac{dx}{dt'}\right)^2 + \left(\frac{dy}{dt'}\right)^2}\, dt', \tag{2}$$

which we approximate as the average swimming speed divided by the helix frequency, or $L_{2D} = 45$ μm. Substituting Eq. (1) into Eq. (2), we can solve for $c = 6.3$ μm. Using this value for c, we can find the 3D trajectory length L_{3D} following:

$$L_{3D} = \int_0^{2\pi} \sqrt{\left(\frac{dx}{dt'}\right)^2 + \left(\frac{dy}{dt'}\right)^2 + \left(\frac{dz}{dt'}\right)^2}\, dt'. \tag{3}$$

We can then estimate the error as $(L_{3D} - L_{2D})/L_{3D}$. Substituting Eq. (1) into Eq. (3), we get an error estimate of 10%.

2 Computation of hydrodynamic drag

To estimate the potential direct hydrodynamic effect of mastigonemes when fluid does not flow between the fibers, we compute the drag coefficients on slender flagella of different cross-sectional geometry using the boundary element method (BEM). Our numerical approach solves the Stokes equations using the completed double-layer boundary integral equation [3], with a completion flow due to a distribution of singularities along the central axis [4].

We estimate the drag coefficient on a straight flagellum without mastigonemes, which we represent as a straight cylindrical rod of length $L = 12\mu$m and radius $r = 0.25$ μm, see **Fig. S2**. The value obtained differs less than 1% from that predicted by the analytical solution $4\pi\mu L/(\ln(L/r) - 0.5 + \ln 2)$, derived by Cox [5] for a motion in the direction perpendicular to the centerline, corresponding to a motion in the Y or Z direction on **Fig. S2**.

This value is compared with a model flagellum with straight rigid mastigonemes, which we represent as a rigid flagellum with an increased effective area. This flagellum is represented as a flattened flagellum of length $L = 12\mu$m, with the same thickness as the cylindrical flagellum $2r = 0.5\mu$m but with a width corresponding to that of the axoneme increased by the two rows of mastigonemes $2(r + l_m) = 2$ μm, see **Fig. S2**. We find the drag coefficient to be significantly larger than for the cylindrical flagellum, with an increase in drag of 32% for a motion in the Y-direction and 50% for a motion in the Z-direction.

3 Supplementary movie captions

Movie S1. Free swimming cell exhibiting large turning rate due to a slip event, where flagella lose synchronous beating. This behavior does not produce the typical forward swimming behavior normally exhibited by the cells, such as in **Fig.2**a, but instead results in the cell turning dramatically without forward progress. The cell turns completely around while staying in the same place. Video is slowed 30X.

Movie S2. Free swimming cell exhibiting large turning rate due to asymmetry in flagella swiping area, where one flagellum swipes a larger area than the other. Specifically, the flagellum on the left swipes a larger area than the one on the right. This results in a larger hydrodynamic thrust generated by the flagellum on the left, which causes the cell to turn to the right. Video is slowed 30X.

Movie S3. One beating cycle of a captured cell from optical tweezers-based velocimetry (OTV) experiments, followed by flow field generated from numerical simulation. Video is slowed 120X.

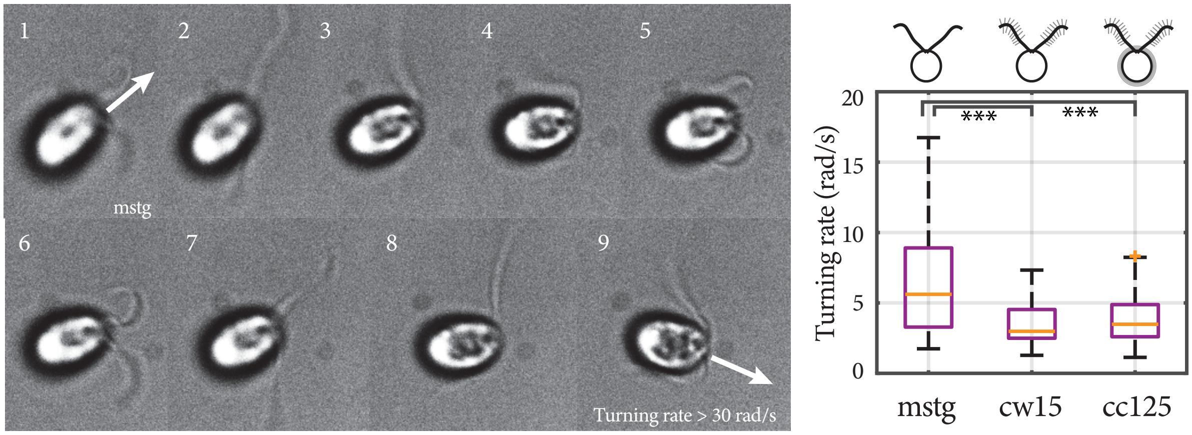

Figure S1: Turning in free swimming observations. (1-9) Image sequence of *mstg* cell during a turning event with a large turning rate. The white arrows represent the cell's heading direction. The time step between the images is 3.3 ms. Comparison of average turning rate for *mstg*, *cw15*, and *cc125* cells from left to right, respectively. Here, $***$ represents $p < 0.001$ following a Kruskal-Wallis One-Way ANOVA statistical test.

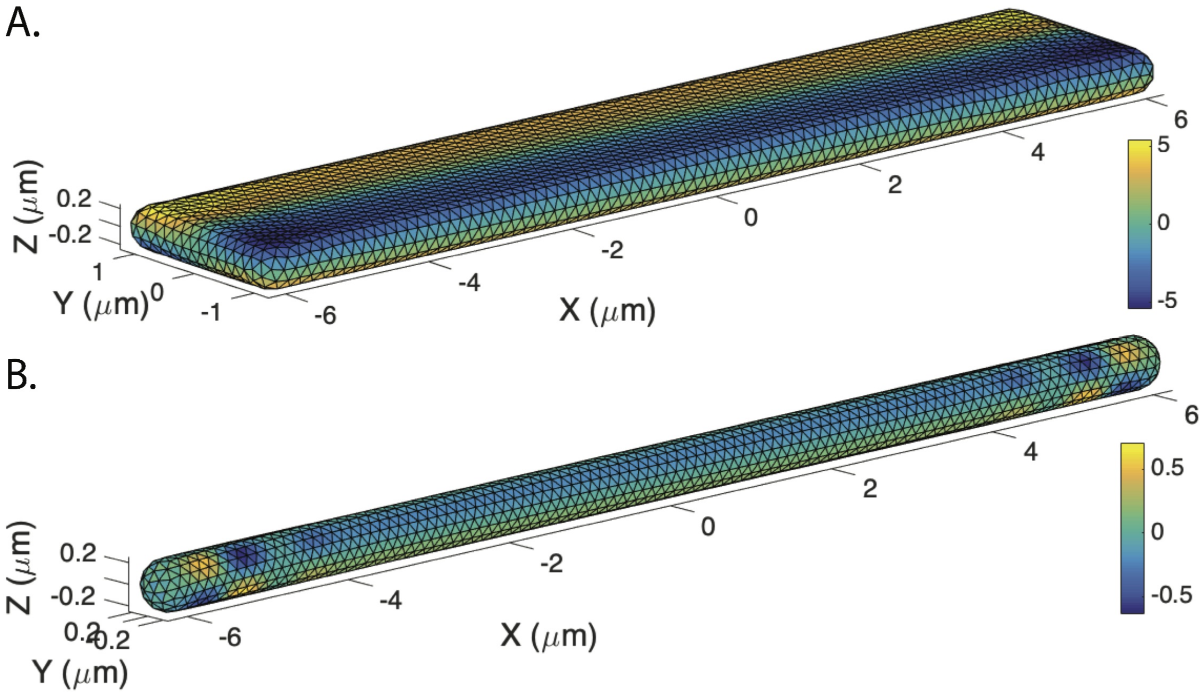

Figure S2: Drag coefficient for different cross-sections. Computation of drag coefficient using BEM on an unstructured triangular mesh. The colormap represents the intensity of one component of the double layer potential. (a) Model flagellum representing a flagellum with two rows of rigid mastigonemes where fluid does not flow between them. (b) Cylindrical flagellum without mastigonemes.

References

[1] Isogai, N., Kamiya, R. & Yoshimura, K. Dominance between the two flagella during phototactic turning in *Chlamydomonas*. *Zoological Science* **17**, 1261–1266 (2000).

[2] Crenshaw, H. C. A new look at locomotion in microorganisms: Rotating and translating. *American Zoologist* **36**, 608–618 (2015).

[3] Power, H. & Miranda, G. Second kind integral equation formulation of Stokes flows past a particle of arbitrary shape. *SIAM Journal on Applied Mathematics* **47**, 689–698 (1987).

[4] Keaveny, E. E. & Shelley, M. J. Applying a second-kind boundary integral equation for surface tractions in stokes flow. *Journal of Computational Physics* **230**, 2141–2159 (2011).

[5] Cox, R. The motion of long slender bodies in a viscous fluid Part 1. General theory. *Journal of Fluid Mechanics* **44**, 791–810 (1970).
